# Extremophilic bacteriorhodopsin from hypersaline salt pan: characterization and photoelectrochemical assessment for potential biosensor applications

**DOI:** 10.3389/fmicb.2026.1805566

**Published:** 2026-06-04

**Authors:** Steffy Joseph, Louis Stanley Abraham, Thirugnanasambandam Rajendran, Dharani Gopal, Dhinakarasamy Inbakandan, Sivakumar Manikandan, Balasubramanian Thangavel, Sumathi Jones, Ratih Setyaningrum, HeeKyung Cho, Ravishankar Ram Mani, Soon Woong Chang, Balasubramani Ravindran

**Affiliations:** 1 Centre for Ocean Research, National Facility for Coastal and Marine Research, Sathyabama Institute of Science and Technology, Chennai, Tamil Nadu, India; 2 Marine Biotechnology Division, National Institute of Ocean Technology, Chennai, Tamil Nadu, India; 3 Centre of Advanced Study in Marine Biology, Annamalai University, Parangipettai, Tamil Nadu, India; 4 Department of Pharmacology and Therapeutics, Sree Balaji Dental College and Hospital, BIHER, Chennai, India; 5 Department of Industrial Engineering, Faculty of Engineering, Universitas Dian Nuswantoro, Semarang, Indonesia; 6 Industry-University Cooperation Foundation, Kyonggi University, Suwon-si, Gyeonggi-do, Republic of Korea; 7 Department of Pharmaceutical Biology, Faculty of Pharmaceutical Sciences, UCSI University, Cheras, Kuala Lumpur, Malaysia; 8 Department of Civil and Energy System Engineering, Kyonggi University, Suwon, Gyeonggi-Do, Republic of Korea; 9 Department of Microbiology, Faculty of arts Science Commerce and Management, Karpagam Academy of Higher Education, Coimbatore, Tamil Nadu, India

**Keywords:** bacteriorhodopsin, extremophiles, haloarchaea, biosensor, photoactive proteins, raman spectroscopy, R-HPLC

## Abstract

**Introduction:**

Salt pans host a diverse microbiome, including specialized extremophilic haloarchaea capable of producing industrially valuable biomolecules, yet remains underexplored extreme environment. Photosensitive bacteriorhodopsin (BR) is a naturally occurring seven helix trans-membrane protein with emerging relevance due to its light driven proton pumping activity, photochemical stability and efficient light to electrical energy conversion.

**Methods:**

In this study, BR was extracted from haloarchaeal strains isolated from the Tuticorin salt pan using the Bead mix method and confirmed by SDS-PAGE. The extracted BR was further characterized using thin-layer chromatography (TLC), reverse-phase high performance liquid chromatography (R-HPLC), and Raman spectroscopy. The Purified BR was evaluated for its photovoltage response and a prototype biosensor was successfully developed.

**Results:**

Haloarchaeal isolate *Halostagnicola larsenii* (TP6) demonstrated a remarkable production yield of 360 mg/L of BR under native minimal saline medium conditions, the notably highest recorded for this native strain to date. This isolate was used for further optimization of BR production involving different carbon and nitrogen sources. Two different protein extraction methods were evaluated, of which the bead mix method proved to be effective, yielding around 48.4 mg/L of BR with 72.7% yield. We examined the morphological and physiological traits of these isolates and confirmed the presence of a 26 kDa protein using SDS-PAGE.

**Discussion:**

The isolated extremophilic haloarchaea have the potential to produce large amount of BR, and the characteristics were similar to those previously reported BR from native strains. The observed photoresponsive ability highlights the potential of BR as a bio-based functional component for food bio-sensing, optical devices, and photoelectrochemical biosensors. Overall, this highlights the feasibility of naturally occurring BR from haloarchaea as a sustainable source of photoactive biomolecules for emerging food biotechnology and sustainable biosensor development.

## Introduction

Salt pans are hypersaline ecosystems that harbor diverse extremophilic microbial communities capable of thriving and functioning under high salinity and elevated ionic stress. Such metabolic, and biochemical adaptations, render these microorganisms as a promising source of novel biomolecules for food and industrial microbiology (Martínez et al., 2022). Among halophilic microorganisms, haloarchaea develop retinal binding proteins (rhodopsins), which transform solar energy into metabolically active ionic gradients, enabling survival under such extreme conditions (Tu et al., 2024). Among microbial rhododpsins, bacteriorhodopsin (BR) remains one of the most studied photoactive membrane proteins, first identified in *Halobacterium salinarum*, where it functions as a light-activated proton pump within the organism’s purple membrane (Gordeliy et al., 2022).

The BR derived from *Halobacterium salinarum*, exhibits remarkable photoelectric properties, making it attractive for applications in optoelectronics, bioenergy, memory storage devices, biosensors, and other industrial applications (Nadella and Hernandez-Baltazar, 2018; Ghosh et al., 2019; Saeedi et al., 2012). Its light-induced conformational changes and stability make it particularly beneficial in food safety and quality control. These biosensors integrate biological recognition elements such as enzymes or antibodies with photoactive membrane protein or optical systems to detect analyte (Singh et al., 2025). Biosensor devices are emerging as one of the foremost relevant diagnostic techniques in the field of food, clinics and environment monitoring purposes due to their rapidity, specificity, ease of mass fabrication, cost effective, and field applicability (Thakur and Ragavan, 2013; Pal et al., 2025).

Despite its potential, large-scale BR production remains a challenge. The conventional source, *Halobacterium salinarum*, requires strict growth conditions and produces relatively low yields, resulting in high production costs (Seyedkarimi et al., 2015). Traditional purification via sucrose density gradient ultracentrifugation (SDGU) was time-consuming and costly, although mechanical methods such as bead mix offer faster, scalable alternatives (Ramanan et al., 2008). For industrial applications, it is essential to identify high-yield BR-producing strains to reduce cost, simplify purification, and enable scalable production (Kashif et al., 2025). Strain-specific genetic and physiological traits significantly influence BR yield, meaning that searching diverse natural habitats increases the likelihood of finding superior producers. Furthermore, while recombinant expression systems (*Esherichia coli*) have been explored (Kahaki et al., 2014; Bratanov et al., 2015), no commercially viable recombinant BR product exists to date. Therefore, explorations of diverse hypersaline habitats remain essential for identifying naturally efficient BR producing strains capable of supporting scalable bioengineering applications.

While BR has been widely studied in *Halobacterium salinarum* isolated from hypersaline ponds (e.g., California, United States), there has been limited exploration of novel native BR-producing haloarchaea from less explored ecosystems like salt pans, especially in India. Previously, several studies from Indian solar slattern have reported the isolation and characterisation of halophilic archaea and bacteria using culture—dependent approaches particularly from Gujarat (Rathakrishnan and Gopalan, 2022; Akolkar et al., 2008), Tamil Nadu (Manikandan et al., 2009a), and along the West coast of India (Raghavan and Furtado, 2000; Kanekar and Kanekar, 2022). In addition, few investigations have explored microbial diversity and pigment producing halophilic archaea using culture dependent approaches from the Kottakuppam solar saltern (Verma et al., 2021) and Marakkanam salt pans (Krishnan et al., 2017; Nagar et al., 2024; Steffy et al., 2022). These investigations primarily focused on microbial diversity analysis or pigment characterization, whereas functional evaluation of BR production and downstream bioelectronics application remains largely unexplored. The Tuticorin salt pans located along the south eastern coast of India represent an extreme hypersaline habitat with fluctuating salinity and intense solar exposure. Previous reports from the Tuticorin salt pans ecosystems have also described microbial diversity and the presence of certain microbial groups, including actinomycetes and other halophilic microorganisms (Jose and Jebakumar, 2012), suggesting that this ecosystem may serve as a potential reservoir of novel extremophiles. However, the present study specifically focusing on the targeted explorations and characterisation BR producing halophiles from the Tuticorin salt pan ecosystem remain limited. This study provides a comprehensive investigation combining isolation, BR optimization, molecular and functional characterization, and evaluation of photocurrent generation toward bio-hybrid biosensor development from haloarchaea obtained from the Tuticorin salt pan ecosystem.

## Materials and methods

### Sample collection and isolation of haloarchaea

Brine sediment samples were collected from the solar salt pan at Tuticorin (8°47’03.6”N 78°07’34.6” E), South-East coast of Tamil Nadu (Supplementary Figure 1). The samples were collected using a sterile spatula in a sterile HDPE (High Density Polyethylene) bag. The temperature and salinity of the salt pan were 36°C and 3.4 M NaCl, respectively. One gram of sediment was weighed, serially diluted, and spread plated on a minimal saline medium. The media composition was presented in Supplementary methodology section 1.1. The plates were incubated at 36°C, 1,500 lux light for 20 days under controlled photoperiod conditions (12:12 h, dark: light). After incubation, the individual pink to red colonies was selected based on the pigment production, size, and morphology for purification by subculturing on minimal saline agar medium. Cell morphology and motility were observed using a phase-contrast optics microscope (Olympus BX41, Japan). Since BR production is primarily associated with haloarchaea, the isolation was designed to selective enrichment and characterization focused on archaeal isolates capable of producing photoactive retinal proteins.

### Screening for bacteriorhodopsin (BR)

The BR concentration in the isolates was determined according to the procedure reported by Sumper et al. (1976). The detailed protocol was mentioned in Supplementary methodology section 1.2. The BR concentration was determined suing the absorbance at 560 nm before (A_1_) and after (A_2_) bleaching. Absorbance values were obtained using a UV-Vis spectrophotometer, with bleaching performed by exposing the sample under light (1,500 lux) for 48 h at RT. The concentration was calculated using the molecular weight of BR (26,000 g/mol) and its molar extension coefficient (63,000/M^–1^ cm^–1^) according to the experiment. The experiments were conducted in triplicates, and the optical density (OD_660_) of the culture strain was adjusted to 2.0. The mean value and standard deviation (SD) were computed to assess the variability within the haloarchaeal strains using the following Formula (1) (Shand and Betlach, 1991) :


$$\mathrm{Bacteriorhodopsin}\left(\mathrm{BR}\right)\left(\mathrm{mg}/L\right)=26,000=\text{[}\frac{\left(\mathrm{A1}-\mathrm{A2}\right)}{63,000}]$$ (1)

### Molecular characterization

The haloarchaeal isolates were identified through 16S rRNA sequencing using Sanger sequencing method. The biomass was extracted from stationary-phase cultures. The genomic DNA extraction was carried out using HiPurA^®^ Bacterial Genomic DNA extraction Kit (MB505 Himedia). The quality and concentration of the extracted DNA were assessed using a Qubit fluorometer (Thermo Fisher Scientific, United States). PCR amplification of the 16S rRNA region was performed using the universal archaeal primers: forward primer 5′-ATTCCGGTTGATCCTGCCGG-3′ (positions 6–25 in *Escherichia coli* numbering) and the reverse primer 5′-AGGAGGTGATCCAGCCGCAG-3′ (positions 1,540–1,521) (Ventosa et al., 2004), targeting approximately 1.5 kb of the archaeal genes. PCR reactions were performed under standard conditions with an annealing temperature of 50°C (Biorad Touchdown 1000 thermal cycler, California) (Supplementary methodology section 1.3). The purified amplified PCR products were submitted for bidirectional Sanger sequencing.

The obtained 16S rRNA gene sequences from Sanger sequencing were manually edited, trimmed and assembled using Bio-Edit software (version 7.7) to remove uncertain bases and low quality regions. Sequence quality analysis was performed based on chromatogram assessment and base calling accuracy. The GC content (%) of each sequence was calculated from nucleotide composition using Bio-Edit software. Homology searches were conducted using the Basic Local Alignment Search Tool (BLASTn) available at the National Center for Biotechnology Information (NCBI) to identify the closely related taxa. The validated sequences were submitted to the NCBI database, and accession numbers were assigned as follows: OQ216882, OQ217021, OR756215, OR756605, OR759139, OR759143, OQ217009, OQ217007, corresponding to isolates TP4, TP1, TP2, TP6, TP8, TP9, TP10, and TP5, respectively.

The evolutionary history of the haloarchaeal isolate was inferred using the Neighbor-Joining method (Saitou and Nei, 1987). The optimal phylogenetic tree was constructed to represent the evolutionary relationship among the isolates. The reliability of the tree topology was evaluated through a bootstrap test with 1,000 replicates (Felsenstein, 1985), and the percentage of replicate trees in which the associated taxa clustered together was indicated at the corresponding branches. Evolutionary distances were calculated using the Kimura 2-parameter method (Kimura, 1980), with values expressed as the number of base substitutions per site. This analysis included 35 nucleotide sequences, considering codon positions and noncoding regions. The total dataset contained 1,498 locations (Tamura et al., 2021), and MEGA11 software was used to conduct evolutionary analysis.

### Optimization studies

A systematic optimization of carbon and nitrogen sources was carried out under different culture conditions to enhance biomass and BR production. Each of the carbon sources (glucose, sucrose, starch), and nitrogen sources (potassium nitrate) was supplemented at different concentrations (0.1, 1, and 5%, w/v) to the minimal saline medium (Supplementary methodology sections 1.4, 1.5) (3.42 M NaCl) and pH adjusted to 7.2. During all the optimization studies 5 mL of haloarchaeal inoculum (OD_660_—1.8) was used. The inoculated medium was incubated under the light (1,500 lux) at RT for 20–25 days. The culture was reached stationary phase at the 14th day of incubation. Optical density (OD) was measured at 660 nm and 560 nm at 2 days intervals to monitor the halo-bacterial growth and Purple membrane synthesis, respectively. Each optimization study was conducted in triplicates, and a medium without culture was used as a negative control.

### Estimation of biomass as dry weight

The biomass dry weight (mg/L) of the cells inoculated in different culture media with different carbon and nitrogen sources was obtained by centrifuging 2 mL (OD_660_- 1.8) of the culture samples at 10,000 rpm for 10 min at 4°C. Aftercentrifugation, the supernatant was discarded, and the pellet in the centrifuge tube was air-dried for 48 h at room temperature using a desiccator. The air dried pellet was weighed, and the dry weight of the biomass was calculated according to the following formula (2):


$$\mathrm{Dry}\,\mathrm{cell}\,\mathrm{biomass}\,\left(\mathrm{mg}/L\right)=\frac{\left(\mathrm{W3}-\mathrm{W2}\right)}{\mathrm{W1}}$$ (2)

Where,

W3 = final weight of the tube along with biomass

W2 = final weight of the control tube

W1 = volume of culture sample

### Extraction and purification of bacteriorhodopsin

#### Standardization of BR protein extraction from halophilic protein

Two different approaches were followed to extract the intracellular membrane protein from halophilic archaea and optimize their yield to maximum. A modified two aqueous phase method developed by Shiu et al. (2013) was used for purifying the BR membrane protein. The BR extraction experiment was carried out in triplicate.

#### Freeze-thaw method for the extraction of BR

In a centrifuge tube, 100 mL (OD_660_ = 1.8) of TP6 culture media was centrifuged at 10,000 rpm for 15 min. After centrifugation, the pellet was collected and completely frozen at -20°C for 1 h. The pellet was then suspended in 5 mL of lysis buffer (100 mM Tris-HCl (pH 7.5), 2M NaCl, 0.5 mM EDTA). The reaction mixture was mixed on a rotary shaker for 1 h at room temperature (RT). The suspended mixture was then frozen again at -20°C for 30 min. After thawing at RT, it was mixed again, and 60 μL of the suspension was removed for total cell extract analysis and centrifuged at 10,000 rpm for 5 min. The pellet was suspended in 1 mL of deionized water, followed by the addition of 60 μL of CHAPS (3-3[(3-Chloamidopropyl) dimethylammonio]-1-propanesulfonate) detergent to aid in membrane protein stabilization and remove the lipid content in the cell lysate. The suspension was homogenized and incubated at 4 °C for 1 h. After thawing to RT, the sample was centrifuged at 12,000 rpm for 10 min. The pellet was suspended in PBS buffer and stored at -20°C until further purification.

#### BR extraction using glass bead mix method

An alternate method for protein extraction from halophilic bacteria was carried out using glass bead mix method. The following steps were performed: The biomass of the TP6 strain was harvested from 100 mL of culture (OD_660_ = 1.8) by centrifugation at 12,000 rpm for 15 min at 4°C. The resultant cell pellet was washed twice with deionized water and frozen for 30 min at -20°C to facilitate cell lysis. Later, the cell pellet was suspended in 5 mL of 100 mM Tris-HCl buffer (pH: 8.0). Disruption was performed using 1 g of glass beads (0.5 mm diameter, Merck) at a bead-to-sample ratio of 0.08:1 (v/v). The mixture was vigorously vortexes at maximum speed for 10 min to disturb the cells mechanically. These standardized conditions consistently yielded reproducible extraction of BR. A detailed optimization of bead size, bead to sample ratio, lysis buffer variations, and mixing parameters has also been performed and will be reported separately. The mixture was then centrifuged at 12,000 rpm for 10 min at 4°C, and the supernatant was collected for cell lysate analysis. The remaining pellet was suspended in 1 mL of deionized water, followed by the addition of 60 μL of CHAPS detergent in the cell lysate. The suspension was homogenized and incubated at 4°C for 1 h. After thawing to RT, the sample was centrifuged at 12,000 rpm for 10 min. The red colored supernatant was collected for further purification.

Purification of this extract was carried out using an aqueous phase method (Shiu et al., 2013). The collected pigmented supernatant was mixed with 10 mL of 24% (w/w) potassium phosphate solution (prepared by dissolving 22 g monobasic potassium phosphate and 2 g dibasic potassium phosphate, pH 8.0) and 10 mL of 24% (w/w) PEG 8000 solution. The mixture was gently mixed and incubated for 30 min for phase separation. The phase separation was achieved by centrifugation at 8,000 rpm for 15 min at 4°C. A distinct red band was formed at the interface of the 2 phases, which was collected and washed with 30 mL of deionized water, followed by centrifugation at 24,000 rpm for 30 min at 4°C. The final pellet was suspended in 5 mL of deionized water and stored at 4°C for subsequent characterization.

The cell lysate from these two extraction methods was used for further BR analysis to analyze the efficiency of the extraction methods and total protein yield. The molecular weight (kDa) of purified BR recovered from the different methods was determined using sodium dodecyl sulfate polyacrylamide gel electrophoresis (SDS-PAGE) of 12% (w/v) acrylamide gel with reference to a medium-range protein marker Ladder (Himedia).

### Analytical methods

#### Bacteriorhodopsin yield and purity determination

The purity of BR extracted from the bead mix and freeze-thaw methods was determined using a UV-VIS spectrophotometer by comparing the absorbance values at 280 nm (aromatic amino acids) (Reinmuth-Selzle et al., 2022) and 560 nm (chromophore) (Mosin and Ignatov, 2014).


$$\mathrm{BR}\mathrm{purity}\left(\%\right)=\frac{\left(\frac{\text{A}\,560}{\mathrm{A280}}\right)}{0.5}$$

The absorbance values at 280 nm (A_280_) and 560 nm (A_560_) represent observations of BR dissolved in deionized water at their respective wavelengths, and 0.5 was a normalization factor as described by Manikandan et al. (2009b) and Morré and Morre (2000). This approach was used to analyze the purity of both the cell lysate and the purified BR produced from two distinct extraction processes. BR yield (%) was calculated as the percentage of BR recovered after purification respect to the initial cured extract concentration (Bollag et al., 1996).


$$\text{Yield}\left(\%\right)=\frac{\mathrm{Purified}\,\mathrm{BR}\,\mathrm{concentration}}{\mathrm{Crude}\,\mathrm{extract}\,\mathrm{BR}\,\mathrm{concentration}}\times 100$$

#### Quantification of bacteriorhodopsin

The concentration of BR obtained through different extraction methods was measured by the hydroxylamine method described by Shand and Betlach (1991). The hydroxylamine bleaching assay represents a classical and widely accepted method for functional quantification of BR in native purple membrane preparations. After extracting the BR solution, 60 μL of 2 M hydroxylamine (pH 7) was added, and the absorbance at 568 nm was immediately measured. During this process, the added hydroxylamine reacts with the Schiff base bond between the retinal chromophore and bacterio-opsin. The mixture was incubated under light (1,500 lux) for 24 h, allowing the bleaching reaction to occur, and the absorbance was measured at the end of incubation. When hydroxylamine interacts with functional BR, the characteristic absorbance at 568 nm disappears. The bleaching process was used only for quantitative estimation did not affect subsequent characterisation or stability of purified BR. The decrease in absorbance at this wavelength was used to estimate the BR content in the different extraction preparations. The BR concentration was measured using the following formula (3):


BR(mg/mL)=(26,800×A-0568A24)568/63000 (3)

where,

A_568_ = Absorbance at 568 nm

S_245_ = Absorbance at 245 nm

26,800—Molecular weight of BR

63,000—Molar extension coefficient

### Characterization

#### UV-Visible spectrophotometer

To confirm the presence of native BR isolated from *Halostagnicola larsenii*, it was initially analyzed using UV-Visible spectrophotometry within the absorbance range of 250–700 nm. The cell lysate and purified samples from both extraction methods was used for the UV-Visible spectrophotometer analysis, with modification to the method described in Cao et al. (2015). A distinct peak characteristic of BR was observed, confirming the presence of the chromoprotein in the purified sample.

#### Thin layer chromatogarphy

Thin-layer chromatography (TLC) was performed to qualitatively confirm the identity of the purified BR by comparison with a commercial BR standard (Sigma Aldrich). TLC analysis was carried out using silica gel 60 F_254_ plate (Merck) as the stationary phase, with acetonitrile as the mobile phase. Purified BR samples (20 μg/mL), and the commercial standard were applied simultaneously and developed for 14 min at RT. The purified sample exhibited a migration patterns and retention factor (Rf value) comparable to the standard BR, supporting the identity of the isolated pigment were comparable with, further validating the identity of the isolated pigment.

#### Raman spectroscopy

Resonance Raman spectroscopy was used to evaluate the influence of the wavelength of excitation on the spectra obtained from the haloarchaea TP6 strain. Raman microprobe (Renishaw, United Kingdom) equipped with a multi-wavelength capability (operating at 532 nm (50 mW) and 785 nm (300 mW) was employed. The analysis was performed with a modification of the method described in Alsafadi et al. (2018). The sample was excited using a 785 nm laser diode with a power of 3 mW, oriented perpendicularly to the sample. The purified BR sample was prepared at a final concentration of 20 μg/mL. Spectral data were acquired with a 20 s exposure time and a single accumulation. The Raman fingerprint region linked to carotenoids encompassed a spectral scan range of 800–1,800 cm^–1^. The Raman spectra provided qualitative confirmation of the BR structural features and chromophore integrity, supporting its functional potential and purity.

#### Reverse-phase high-performance liquid chromatography (R-HPLC) analysis

The purity and elution profile of the isolated BR protein were examined using reverse-phase high-performance liquid chromatography (R-HPLC). The chromatographic separation was performed on a C18 reverse-phase column (5 μm particle size, 4.6 × 250 mm, Shim-pack solar, Shimadzu) connected to a Shimadzu LC-20 AD RF-HPLC system. Equipped with a dual pump, the absorbance was monitored at 280 nm using a photodiode array detector (PDA) with modification of the method described in Yuan et al. (2018). The mobile phase consisted of acetonitrile. Isocratic elution was employed with acetonitrile concentration maintained at 99% (v/v) throughout the run. The flow rate was set at 1 mL/min, and the column temperature was maintained at room temperature (25°C). The total run time was 60 min. A 20 μL aliquot of the purified BR protein sample and BR standard (Sigma Aldrich) was injected for each run. The retention time and peak area were recorded and analyzed using Labtech software. To ensure the accuracy and reproducibility of the analysis, each sample was injected in triplicate. The retention time and peak area values were averaged, and the mean values were used for further interpretation.

#### Design and assembly of bacteriorhodopsin based biosensor

Indium tin oxide (ITO) glass substrates were initially cleaned using a phosphate-free detergent, rinsed thoroughly with isopropanol, and dried at RT. A gold layer (Au) with approximate thickness of 500 nm was deposited onto the cleaned ITO glass by sputter coating. A polyvinyl alcohol (PVA) solution was prepared separately and uniformly coated over the gold-coated ITO substrate. The assembly was left to dry under ambient conditions for 12 h. The purified BR was carefully oriented onto ITO/Au/PVA surface and allowed to dry completely. The sandwich-structured device, comprising ITO, Au, PVA and BR layers, was incubated for 1 h to ensure proper film stabilization. The photo voltage measurement was performed using a digital voltmeter. The device response was first recoded under dark phase for 0–5 min, followed by light illumination phase (1,500 lux) for 5–60 min. Photovoltage responses were recorded continuously to evaluate the photoelectrical performance of the assembled bio-hybrid system.

#### Statistical analysis

The data obtained from the optimization study were analyzed using two-way ANOVA (SPSS version 22) to evaluate the different treatments of carbon, nitrogen sources, and concentrations, as well as their interactive effect on haloarchaeal growth and BR production. The assumptions of normality and homogeneity of variances were verified using Shapiro-Wilk and Levene’s tests, respectively, before analysis. The significance level was set at *P*<0.05, and the effect sizes were reported using Partial Eta Squared (η^2^) to determine the proportion of variance explained by each factor. *Post-hoc* pairwise comparisons were carried out with Bonferroni corrections when significant main effects were detected. All the experiments were conducted in triplicates.

## Results

### Isolation of haloarchaea from salt pan

A total of twenty-two pigmented colonies displaying various colors, notably yellow, orange, pink, and red, were initially acquired using spread plating of hyper saline sediment samples from Tuticorin salt pan (Supplementary Figure 2a). As BR synthesis is often associated with red to pink pigments haloarchaea, colonies with red to pink pigmentation were deliberately picked for further study. Based on pigmentation features and colony morphology, eight typical isolates selected and sequentially labeled as TP 1, TP 2, TP 4, TP 5, TP 6, TP 8, TP 9, TP 10 (Supplementary Figure 2b). The distribution of colony pigmentation observed during primary screening is summarized as follows: 4 yellow, 5 orange, 7 pink, 3 red colonies.

All examined isolates exhibited distinctive pigmentation ranging from light pink to red, indicating of haloarchaeal carotenoid and retinal-associated pigments. Microscopic examination revealed primarily coccid cell morphology, whereas isolate TP6 showed pleomorphic rod- shaped cells. Gram staining revealed predominantly Gram-negative like features, while isolate TP5 exhibited a gram positive like response, a feature occasionally observed among haloarchaea due to variations in cell envelope composition. All selected isolates were non-motile under the tested conditions. Subsequent optimization studies revealed that these isolates grow optimally at a temperature of 24°C, pH 7.2, and salinity of 3.43 M NaCl or 20% (w/v) of NaCl.

### Quantification and comparative analysis of bacteriorhodopsin production

**FIGURE 1:**
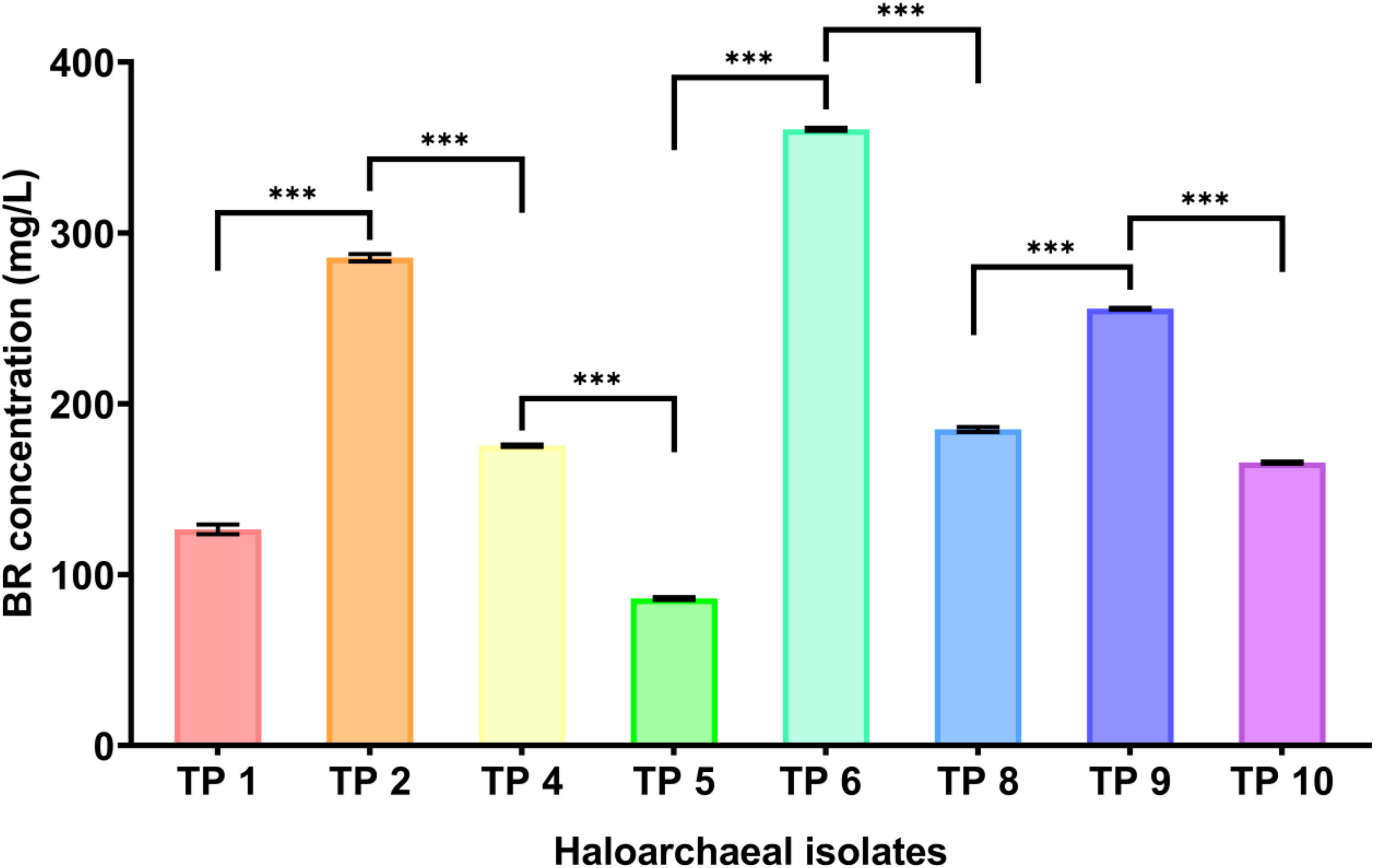
Comparative BR production among haloarchaeal isolates. Data are indicate as mean ± standard deviation (SD) (*n* = 3) and the statstical significance was evaluated using two way ANOVA followed by post hoc analysis, where ***p <0.001 indicate highly siginificant difference between isolates.

The BR concentrations of haloarchaeal isolates ranged from 86.0 ± 0.8 to 360.65 ± 0.9 mg/L (Mean ± SD) (Figure 1). The standard deviation (SD) remained consistently low across all strains ( ± 0.49 to ± 2.8 mg/L). These consistent values reinforce the reliability and stability of BR production in the isolated strains. Eight isolates tested, TP6 exhibited the highest BR concentration 360.65 ± 0.9 mg/L followed by TP2, TP8, and TP9 (Table 1). Conversely, isolates TP1, TP4, TP5, and TP10 exhibited moderate BR concentrations, while the remaining strains showed lower production levels. TP6 isolate exhibited the highest BR yield, stable pigmentation and steady growth performance under optimized culture conditions irrespective of the eight isolates examined. Therefore, this isolate was selected as the primary organism for further optimization, downstream BR extraction, purification, characterization, photocurrent application studies.

**TABLE 1:** Morphological and biochemical characteristics of halophilic archaeal isolates, including taxonomic identification, gram reaction, pigmentation, and bacteriorhodopisn concentration (mg/L; mean ± SD).

Isolates name	Taxonomic identification	Pigmentation	Gram ±	Morphology	Bacterio rhodopsin concentration (mg/L)	Motility
TP 1	*Halococcus agarilyticus*	Light pink	–	Cocci	126.5 ± 2.8	Non-motile
TP 2	*Halophilic archaeon*	Light orange	–	Cocci	285.5 ± 2.1	Non-motile
TP 4	*Haloalkalicoccus paucihalophilus*	Light pink	–	Cocci	175.5 ± 0.7	Non-motile
TP 5	*Haloalkalicoccus paucihalophilus*	Orange	+	Cocci	86 ± 0.8	Non-motile
TP 6	*Halostagnicola larsenii*	Pink	- Pleomorphic	Rod	360.65 ± 0.9	Non-motile
TP 8	*Halococcus salifodinae*	Orange-red	–	Cocci	185 ± 1.41	Non- motile
TP 9	*Halococcus* sp.	Red	–	Cocci	255.65 ± 0.49	Non-motile
TP 10	*Haloalkalicoccus paucihalophilus*	Pink	–	Cocci	165.6 ± 0.56	Non-motile

### Molecular characterisation of isolates

**FIGURE 2:**
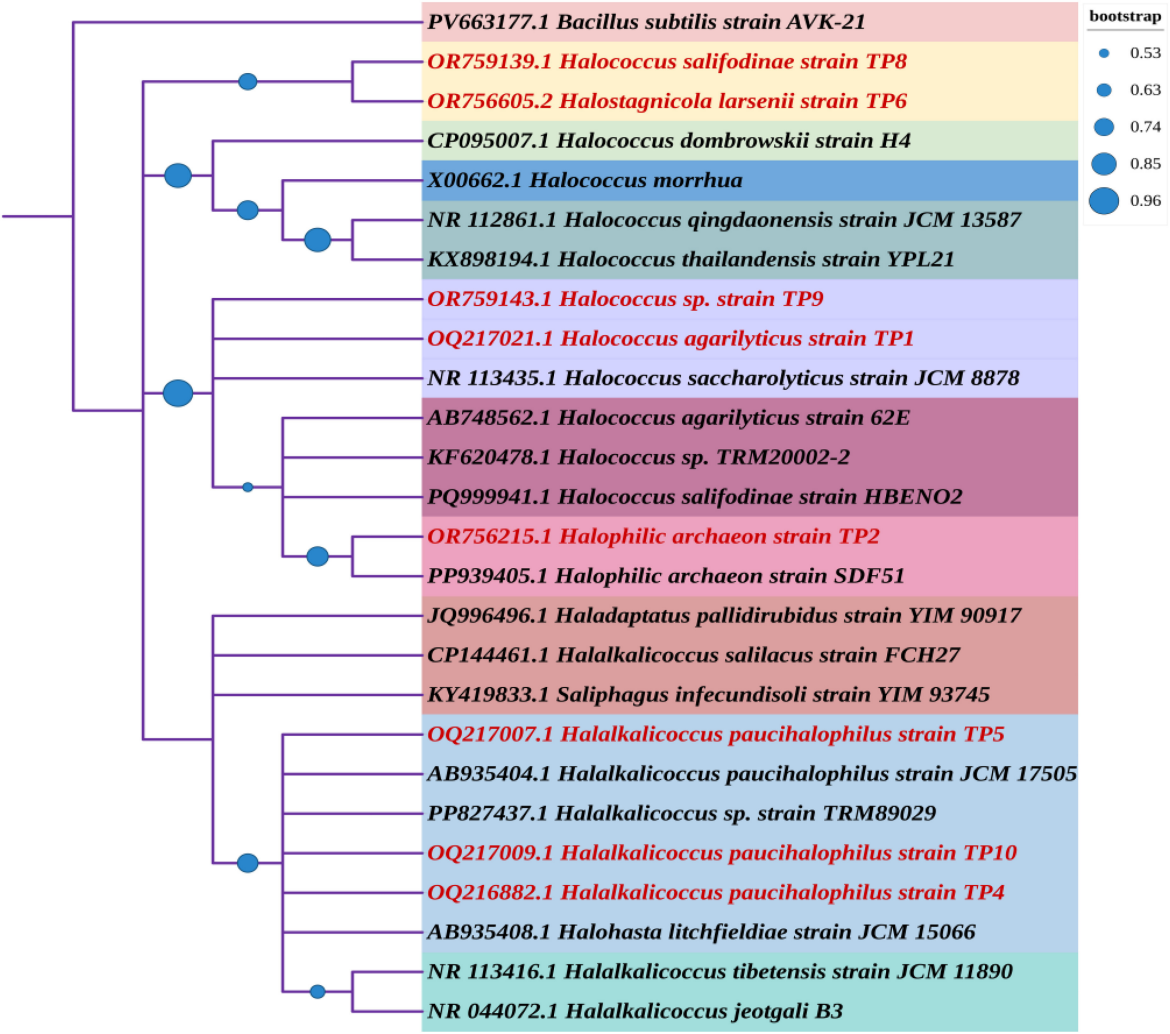
Phylogenetic tree of the isolated haloarcheal strains from tuticorin salt pan. The isolated strains were highlighted in red color.

The phylogenetic analysis based on 16S rRNA gene sequences revealed that the isolates belong to three haloarchaeal genera. Isolates TP1, TP2, TP8, and TP9 clustered within the genus *Halococcus*, whereas isolates TP4, TP5, and TP10 were affiliated within the genus *Haloalkalicoccus* and the isolate TP6 formed a distinct clade closely related to *Halostagnicola*. The sequence similarity analysis showed that isolate TP1 and TP9 exhibited 99–100% similarity to *Halococcus* spp., while TP6 demonstrated 98.15% similarity to *Halostagnicola larsenii*. Additionally, TP5, TP10, and TP4 showed 98–100% similarity to *Halalkalicoccus* sp., and TP8 revealed 94.03% similarity to *Halococcus salifodiniae*. The 16S rRNA sequence of TP2 was closely associated with an uncultured *Halophilic archaeon*, sharing 99% similarity. Phylogenetic analysis based on the 16S rRNA gene sequence further confirmed the taxonomic placement of the isolates, with TP6 clustering closely proximity to *Halostagnicola larsenii* and clearly separated from other haloarchaeal genera (Figure 2). The study isolates were highlights in red color, whereas reference strains were represented in black color in the phylogenetic tree.

High quality sequences ranging from 720 to 1,125 bp were obtained. The GC content of the isolates ranged between 57.71 and 60.56%. Detailed sequencing statistics were summarized in Table 2.

**TABLE 2:** Sequencing data and quality assessment.

Strain ID	Sequence length (bp)	GC content (%)	QC (%)	GenBank accession no.
TP1	1,009	60.56	75.86	OQ217021
TP2	997	60.78	74.40	OQ217021
TP4	1,125	58.49	87.20	OQ216882
TP5	926	58.86	69.10	OQ217007
TP6	1,135	57.71	87.20	OR756605
TP8	988	58.40	74.84	OR759139
TP9	890	60.45	67.42	OR759143
TP10	721	58.80	55.03	OQ217009

### Optimization of culture conditions for growth and BR production

#### Effect of carbon source on growth and BR production

**FIGURE 3:**
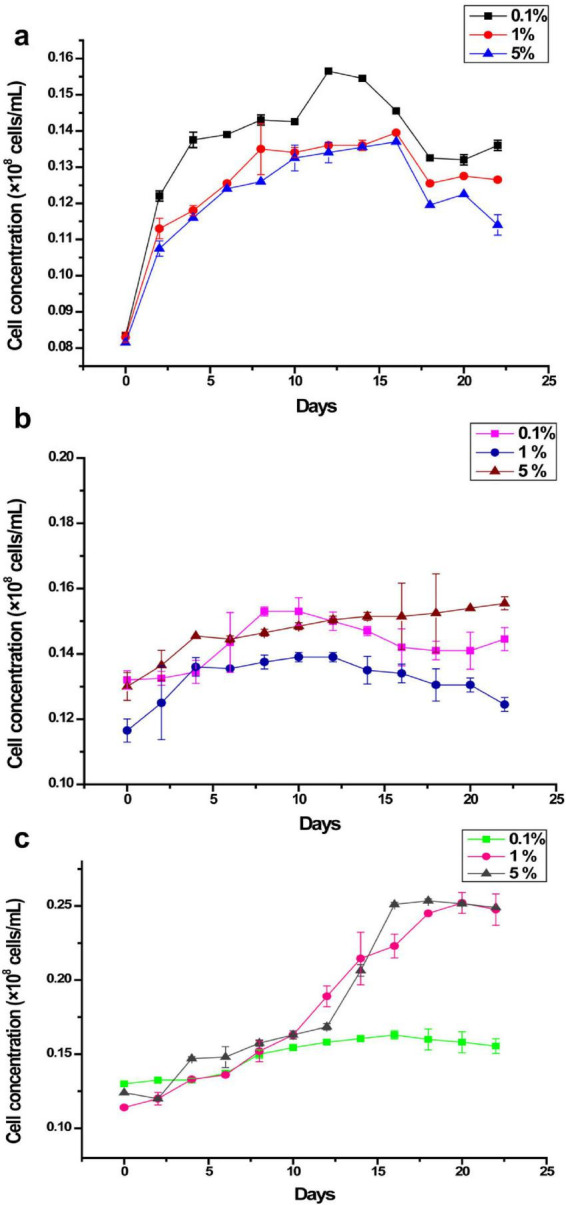
Growth curve showing the cell concentration ( × 10^8^ cell/mL) of TP6 strain in minimal saline medium supplemented with different concentrations of carbon sources. **(a)** growth curve with variying glucose concentrations, **(b)** growth curve with varying sucrose concentrations, and **(c)** growth curve with varying starch concentrations. Error bars indicate standard deviation (SD) from three independednt experiments. The results indicate that supplementation with 0.1 and 5% sucrose significantly enhanced the growth of TP6 strain.

**FIGURE 4:**
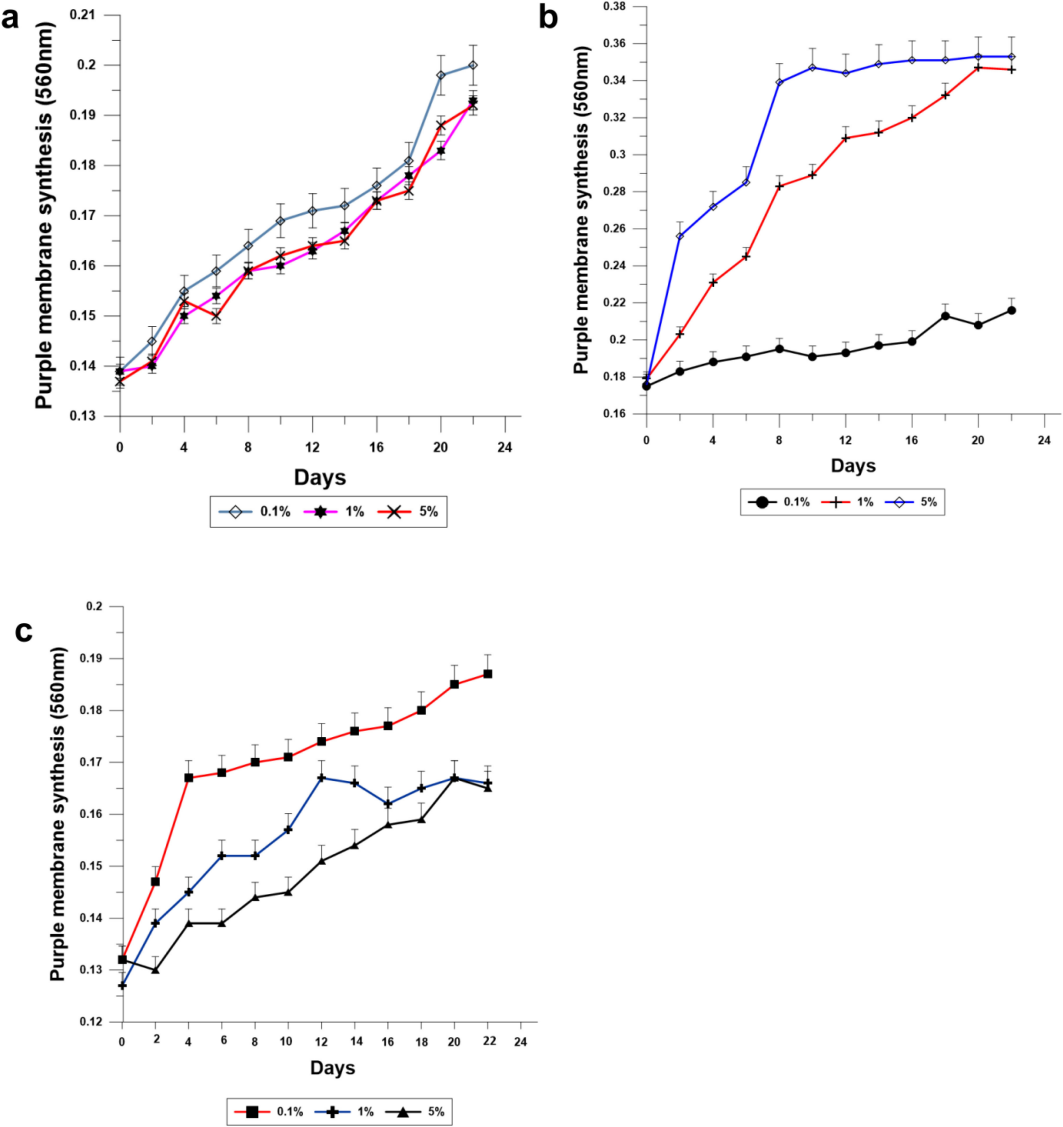
Absorbance measurements at 560 nm representing purple membrane protein synthesized by TP6 strain cultured in minimal saline media supplemented with different carbon sources at varying concentrations **(a)** purple membrane synthesis in medium with sucrose **(b)** purple membrane synthesis in medium with starch **(c)** purple membrane synthesis in medium with glucose. Error bars indicate standard deviation (SD) from three independent experiments.

**FIGURE 5:**
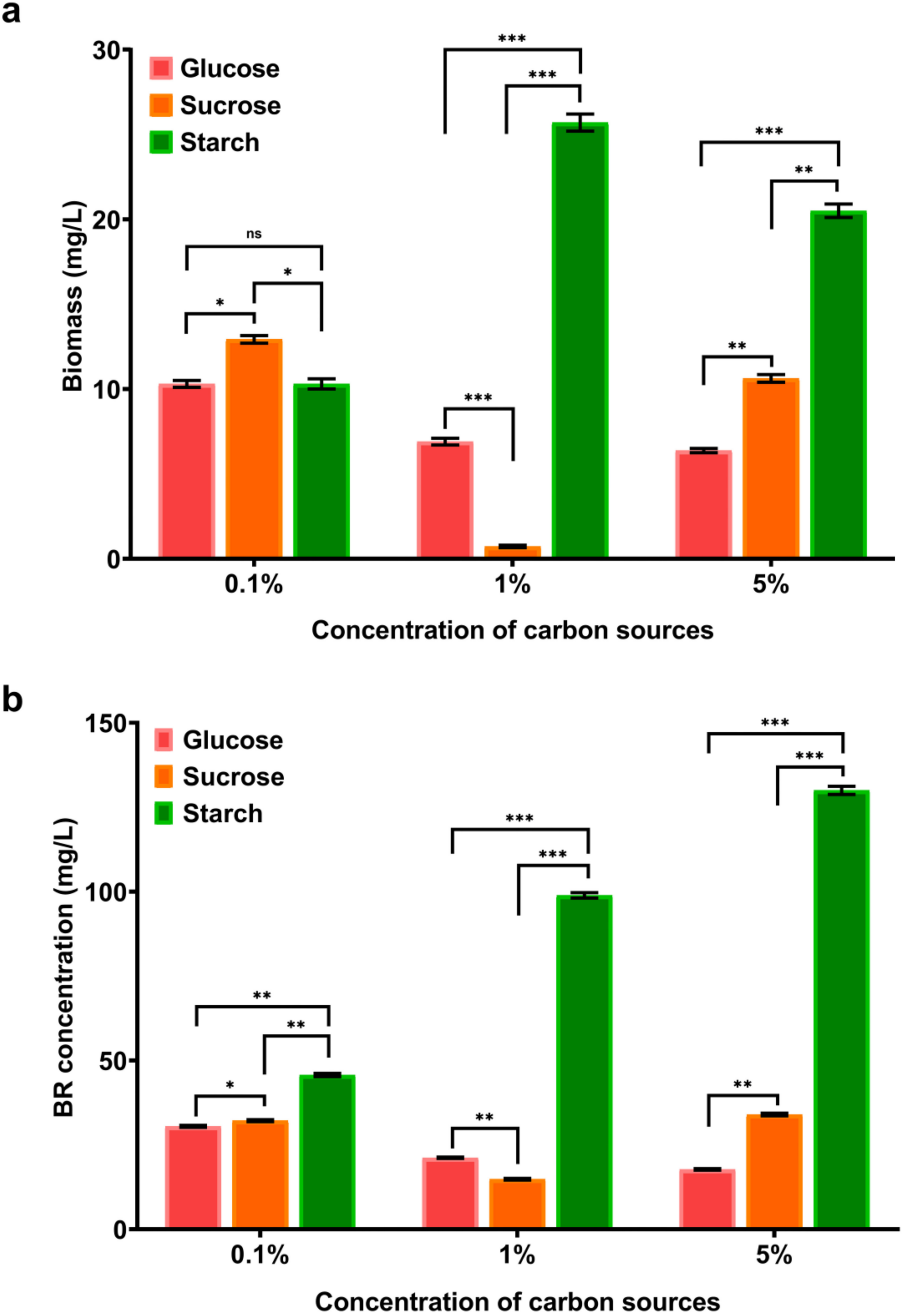
**(a)** Biomass yield (mg/L) of TP6 strain grown in minimal saline media supplemented with different carbon sources at various (0.5, 1, and 5%) concentrations. **(b)** The yield of BR concentration (mg/L) of TP6 Strain under the same conditions with varying concentrations of carbon sources. Error bars indicate standard deviation (SD) (*n* = 3) and the significance was evaluated using two way ANOVA followed by *post hoc* analysis, where *p < 0.05, **p < 0.01, ***p < 0.001, and ns indicates no significance.

The growth rate and BR production varied with different carbon sources at different concentrations. A moderate increase in growth and BR production was observed in *Halostagnicola larsenii* supplemented with 0.1% glucose as carbon source (Figures 3a, 5b). Though a similar growth rate was observed with sucrose at all tested concentrations, a modest increase in BR production was observed only with 0.1 and 5% sucrose as carbon source (Figures 3b, 5b). Alternatively, starch at 1 and 5% significantly promoted both cell growth and BR production, whereas, 0.1% starch showed no notable effect (Figures 3c, 5b). Among all carbon sources, *Halostagnicola larsenii* yielded high BR and minimal saline medium supplemented with 5% starch, and high biomass in 5% sucrose, yet both values remained substantially lower than native control strain (Figures 4a, 5b). Although carbon concentration significantly affected growth (*F* = 10.09, *p* = 0.005, η^2^ = 0.692), it did not significantly influence BR yield (*F* = 2.30, *p* = 0.156, η^2^ = 0.338) according to this optimization study. The statistical two-way ANOVA results are shown in Supplementary Table 2. A significant interaction between carbon source and concentration was observed for bacterial abundance (*F* = 37.24, *p* = 0.162, η^2^ = 0.484). The statistical analysis confirmed that the observed variations in TP6 growth and BR production across different carbon sources were statistically significant (*p* < 0.001) and BR production (*p* = 0.005), whereas variations in concentration within the same carbon source did not significantly influence BR yield (*P* > 0.05). Under controlled conditions without carbon supplementation, *Halostagnicola larsenii* yielded the maximum BR concentrations of 360.65 ± 0.9 mg/mL confirming that native nutrient limiting conditions inherently promote BR synthesis (Supplementary Table 1). The optimization of the carbon source was performed to evaluate whether exogenous carbon availability influences biomass formation and BR synthesis in *Halostagnicola larsenii*. However, BR concentration consistently reduced in supplementation compared to native minimal saline medium conditions indicating that BR production is independent of exogenous carbon availability. Reduced metabolic utilization efficiency rather than lack of carbon impact may be the cause of irregular trend observed with sucrose.

#### Effect of nitrogen sources on growth and pigment production

**FIGURE 6:**
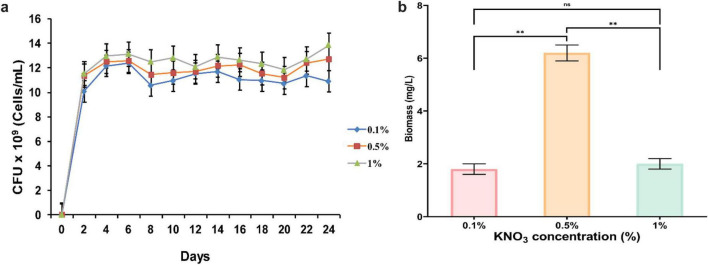
**(a)** Growth curve showing of TP6 strain over 24 days in minimal saline medium supplemnted with different concentrations of nitrogen source (KNO_3)_, **(b)** Biomass concentration (mg/mL) of TP6 strain after the growth period under the same conditions. Error bars indicate standard deviation (SD) (*n* = 3) and the significance was evaluated using two way ANOVA followed by *post hoc* analysis, where *p < 0.05, **p < 0.01, ***p < 0.001, and ns indicates no significance.

The nitrogen source KNO_3_ has no statistically significant impact on both biomass and BR yield of the *Halostagnicola larsenii* (*p* > 0.05) (Figures 6a,c). The addition of a nitrogen source resulted in lower biomass yield compared to the control media with no added nitrogen source (Figure 6). Among different concentrations of nitrogen supplemented with growth medium, maximum biomass (5.5 mg/L) was achieved in the medium supplemented with 0.5% KNO_3,_ though this remained low compared to yields achieved without inorganic supplementation.

**FIGURE 7:**
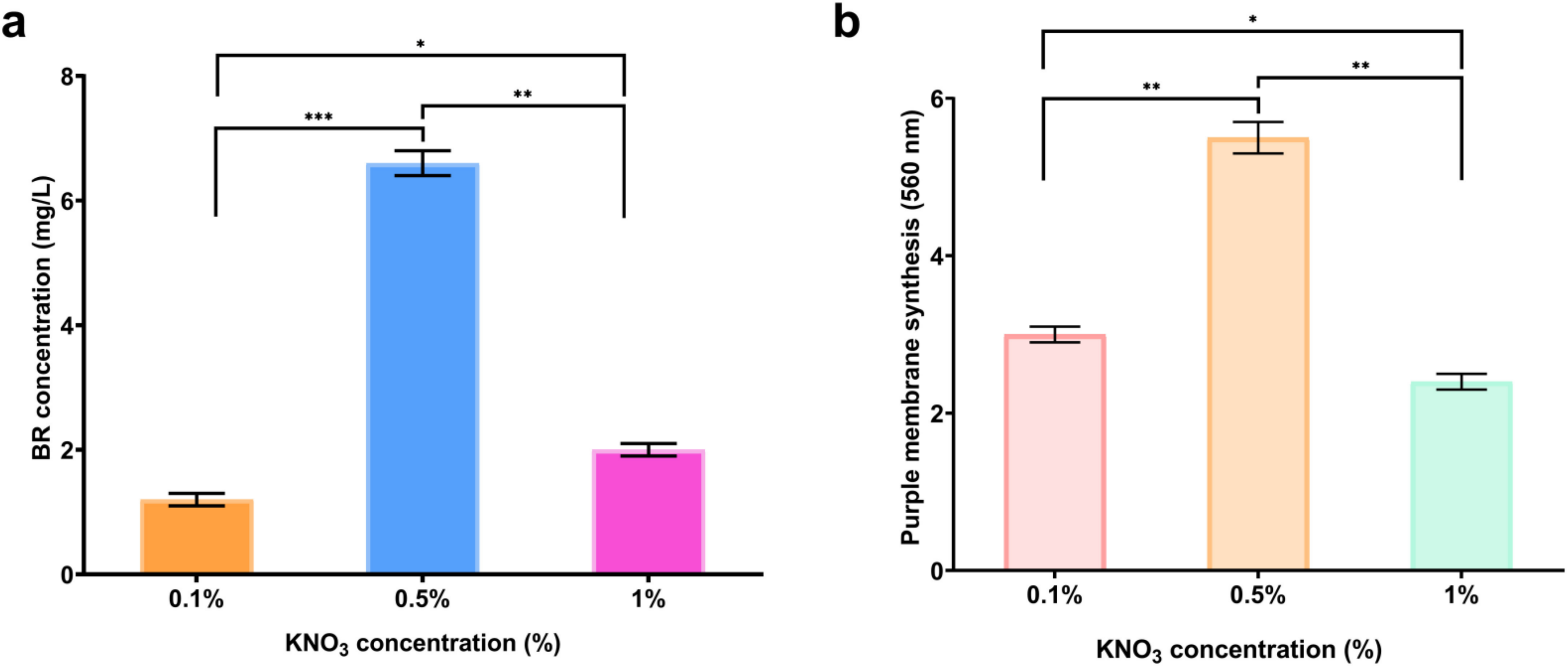
**(a)** The BR concentration (mg/L) of TP6 Strain under the same conditions with varying concentrations of nitrogen source. **(b)** Purple membrane Synthesis measured by absorbance at 560 nm in cultures grown with varying KNO_3_ concentrations Error bars indicate standard deviation (SD) (*n* = 3) and the significance was evaluated using two way ANOVA followed by post hoc analysis, where *p < 0.05, **p < 0.01, ***p < 0.001, and ns indicates no significance.

The two way ANOVA showed that the nitrogen source had a significant impact on bacterial abundance (*F* = 245.656, *P* < 0.001, η^2^ = 0.938) (Supplementary Table 2) and, it did not show any notable independent effect on BR yield (*P* = 0.187). However, the interaction between nitrogen source and concentration was significant for both growth (*F* = 36.48, *P* < 0.001, η^2^ = 0.925) and BR yield (*F* = 35.67, *p* < 0.001, η^2^ = 0.936), indicating that BR production depends on the specific pairing of nitrogen type and concentration rather than either factor alone. Simultaneously, BR concentration reduced significantly across all nitrogen supplemented conditions compared to control, confirming that nitrogen enrichment negatively impacts BR biosynthesis in *Halostagnicola larsenii* (Figure 7 and Supplementary Table 1).

#### Extraction, purification and yield of BR

The bead mix (BM) method combined with a modified lysis buffer yielded supreme extraction efficiency for BR from *Halostagnicola larsenii* compared to the conventional freeze-thaw (FT) technique. The crude cell lysate obtained from the BM approach exhibited a superior BR concentration (66.6 mg/L) compared to FT approach (54.4 mg/L). The initial purity of BR in crude extract was low with value of 13.2% for BM and 10.8 % for FT extraction method.

**FIGURE 8:**
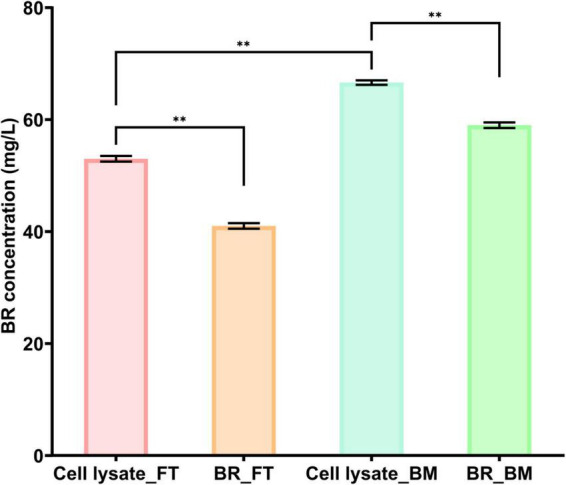
Comparison of BR extraction methods from TP6 starin. Comparison of BR concentration (mg/L) obtained from the different extraction methods. Error bars indicate standard deviation (SD) (*n* = 3) and the significance was evaluated using two way ANOVA followed by *post hoc* analysis, where **p < 0.01 indicates moderalty significance in between the methods.

Following aqueous two-phase purification, the BR concentration was reduced to 48.4 mg/L for the BM method and 36.8 mg/L for the FT method, reflecting removal of non-target cellular components (Figure 8 and Table 3). The purification substantially improved BR purity to 61% for the BM method and 53% for the FT method. The overall BR yield after purification was determined as 72.7% for the BM approach and 67.6% for the FT method, showed improved recovery efficiency using mechanical disruption. The observed improvement results from the combined effect of mechanical disruption and detergent assisted extraction. The delipidated BR preparations displayed a prominent protein band at approximately 26 kDa in the SDS-PAGE gel analysis, confirming successful enrichment of BR (Supplementary Figure 3).

**TABLE 3:** Comparative analysis of BR concentration, purity, total yield, and fold purification obtained from different extraction and purification steps using bead mixing (BM) and freeze thaw (FT) methods.

Preparation method	BR concentration (mg/L)	BR purity (%)	Yield (%)	Fold purification
Cell lysate-BM	66.6	13.2	–^a^	1^b^
Cell lysate-FT	54.4	10.8	–^a^	1^b^
Purified BR-BM	48.4	61	72.7	4.62
Purified BR-FT	36.8	53	67.6	4.90

*^a^*Not applicable: yield was not measured from crude cell lysate fractions.

*^b^*cell lysate fractions were set as the reference value (1.00) for fold purification calculation.

**FIGURE 9:**
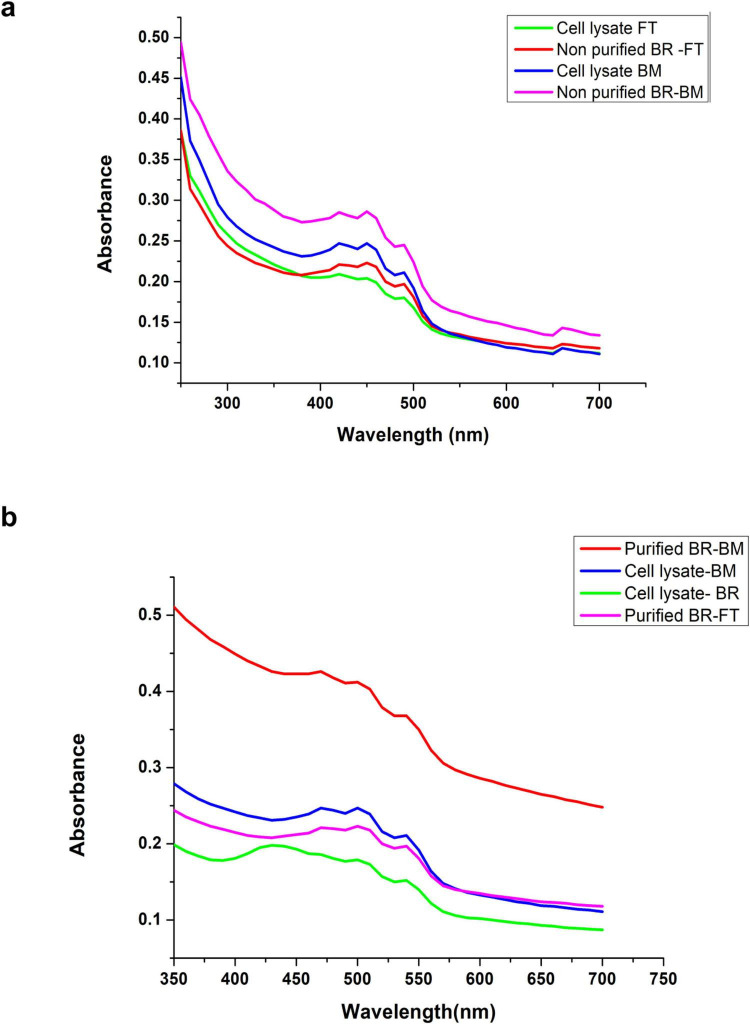
UV-Vis absorption spectra of BR obtained from different extraction processes **(a)** UV-Vis spectra of cell lysate and non-purified BR. **(b)** UV-Vis absorption spectra of purified BR extracted using both extraction methods.

The UV–Vis absorption spectra of the cell lysate containing BR from the BM and FT methods are shown in Figure 9a. The cell lysate exhibited very low absorbance at 560 nm but a strong peak at 280 nm. Based on the ratio of absorbance at 560 nm (indicative of BR) to 280 nm (representing total protein and aromatic amino acids), the BR purity of BM cell lysate was calculated to be 13.2%. In comparison, the FT cell lysate extracted samples displayed broader, less distinct absorbance profiles. Based on the ratio of absorbance at 568–280 nm, the BR purity in BM preparation was determined to be 61%. The purified BR from the BM method displayed a strong peak near at 550 nm, indicating a high yield and purity (Figure 9b). It also showed maximum peaks at 460 nm and 510 nm, attributed to the presence of bacterioruberin. To confirm the presence and structural intergrity of BR, we validate its purity and reduce interference from co-extracted pigments through RP-HPLC chromatography and Raman spectroscopy.

### Characterization of bacteriorhodopsin

#### Thin layer chromatography

The identity of the native BR isolated from *Halostagnicola larsenii* TP6 was further confirmed using thin-layer chromatography (TLC) (Supplementary Figure 4). The retention factor (Rf) value of the native BR was recorded at 0.731, closely matching the Rf of the standard, which was 0.716.

#### Raman spectroscopy analysis

**FIGURE 10:**
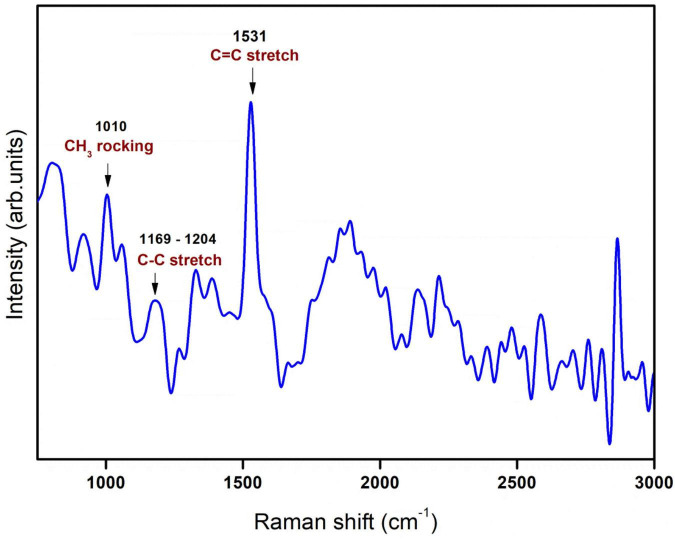
Raman resonance spectrum of purified BR extracted from TP6 strain. The spectrum shows characteristic vibrational peaks at 1,153 and 1,509 cm^– 1^, corresponding to major structural features of the BR chromophore.

Resonance Raman spectroscopy was used to determine the structural characteristics of BR extracted from the *Halostagnicola larsenii*. The spectra obtained revealed prominent peaks at 1,010, 1,170, 1,204, and 1,530 cm ^–1^ (Figure 10). In the purified BR, the C = C (V1) stretching mode was observed at 1,530 cm^–1^, while the C–C stretching appeared with much weaker intensity in the 1,170–1,204 cm^–1^ range. These characteristic features attributable to BR in the TP6 strain. Additionally, vibrations in the 1,010 cm^–1^ area have been linked to in-plane rocking modes of CH groups connected to the polyene chain, coupled with C–C bonds. The pigment’s red appearance intensifies with an increasing conjugated (C = C) bonds, resulting in a longer wavelength of absorbed light. The length of the BR chain is highly associated with the Raman shift (cm^–1^) of the V1 band.

#### Reverse-phase HPLC analysis of purified BR

**FIGURE 11:**
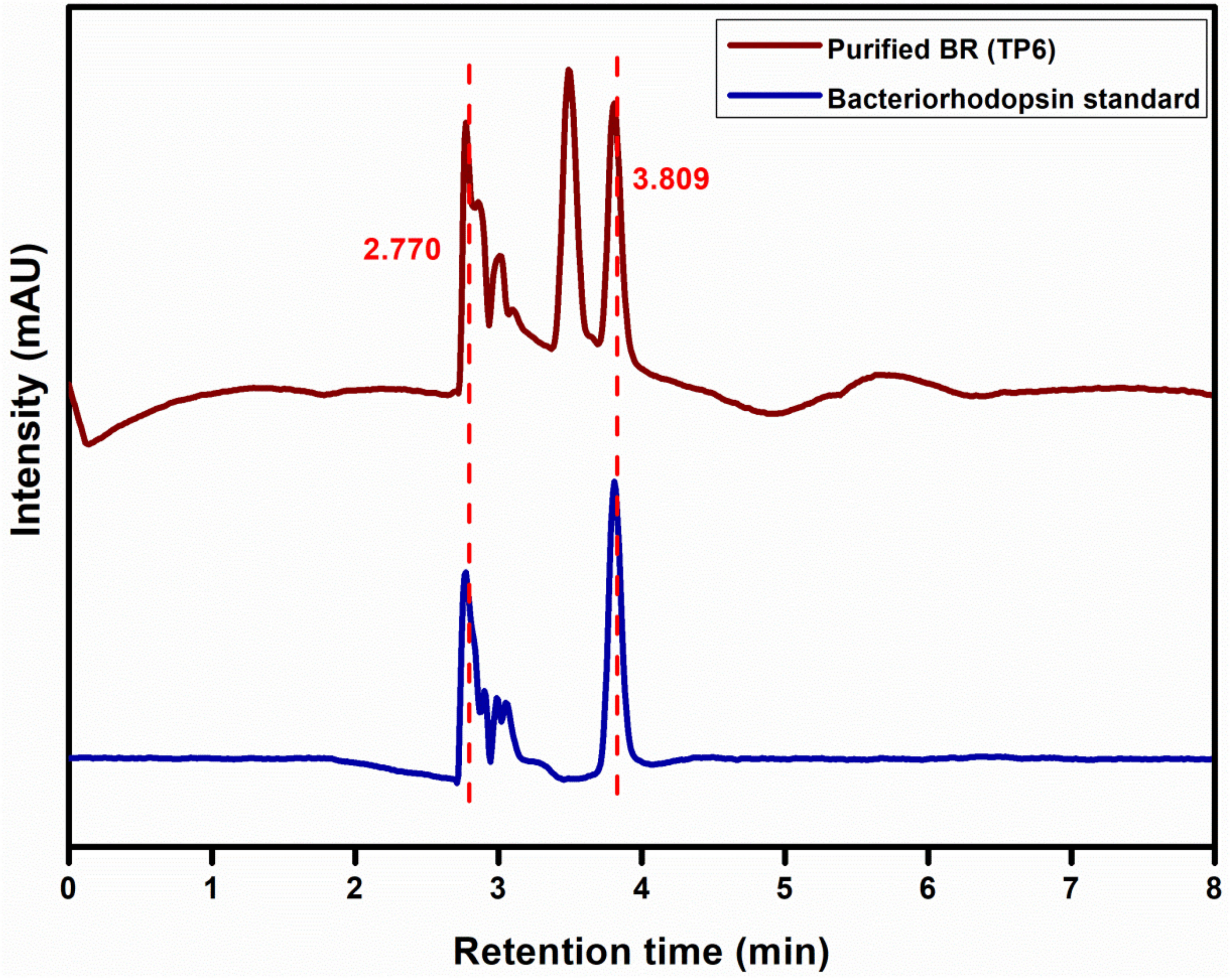
Reverse phase high performance liquid chromatography (RP-HPLC) chromatogram of bacteriorhodopsin (BR) at 280 nm. The chromatogram shows the elution profiles of the standard BR protein and the native BR isolated from *Halostagnicola larsenii* TP6. This chromatorgram higlights the purity of the extracted BR compared with standrad bacteriorohodpsin.

The purity and elution patterns of the standard BR protein and the BR isolated from *Halostagnicola larsenii* were evaluated by reverse–phase HPLC (RP-HPLC) at 280 nm using an isocratic elution system. The mobile phase consists of concentrated acetonitrile maintained under isocratic conditions. The elution profile was monitored at 280 nm, targeting the aromatic amino acid residues of the protein. The RP-HPLC chromatogram shown in Figure 11, exhibited three distinct peaks within the retention time window of 2.5–4.5 min. The standard BR chromatogram of Figure 11, showed two strong and well-resolved peaks at retention times of 2.8 and 3.5 min, with a solid baseline and few extra peaks, indicating high purity BR (about 95–98% based on peak area analysis). In comparison, the chromatogram of the BR extracted from *Halostagnicola larsenii* revealed corresponding significant peaks at 2.85 and 3.5 m, demonstrating the existence of BR in the native preparation. However, many minor peaks were detected between 2.5 and 4.5 min, accompanied by slight baseline changes, indicating the presence of mixed micelles of proteins or other lipids in the crude membrane extract. The major BR peak at 3.5 min accounted for 75–80% of the entire peak area, indicating a significant concentration of monomeric delipidated BR in the preparation, but with detectable mixed micelles of protein or lipids. These peaks are postulated to correspond either to heterogeneous molecular forms of BR, such as partially denatured fragments or retinal-protein complexes dissociated during the denaturing acetonitrile elution.

#### Photoelectrochemical characterization of the BR based biosensor assembly

**FIGURE 12:**
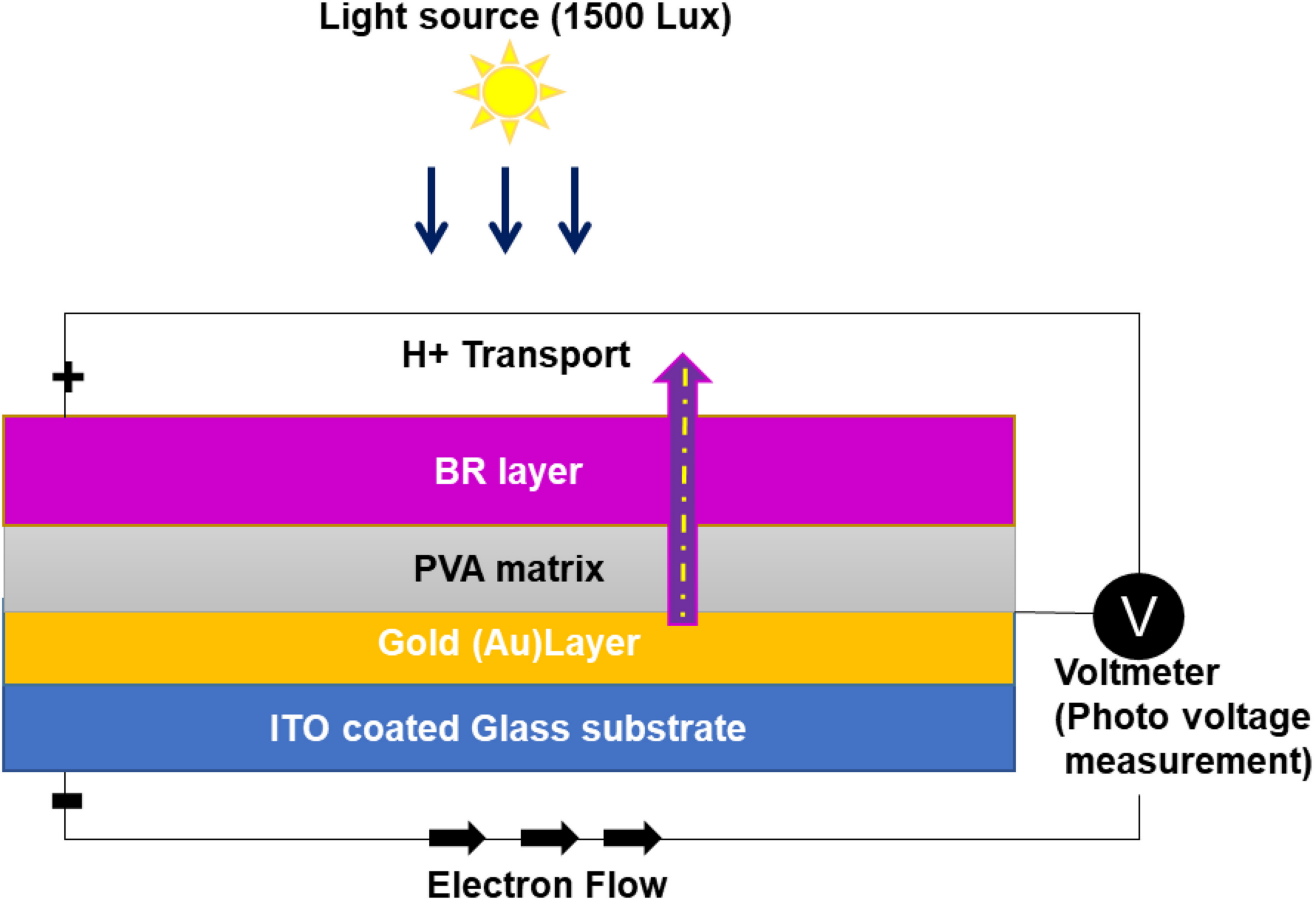
Schematic representation of the fabricated ITO/Au/PVA/BR based biosensor device. Illustration of the layered architecture of the fabricated biohybrid system comprising indium tin oxide (ITO) coated glass substrate, sputter deposited gold (Au) conductive layer, polyvinyl alcohol (PVA) matrix for protein immobilization and BR photoactive layer. Photo-voltage generation was measured across the device under dark and light illumination conditions using an external voltmeter.

**FIGURE 13:**
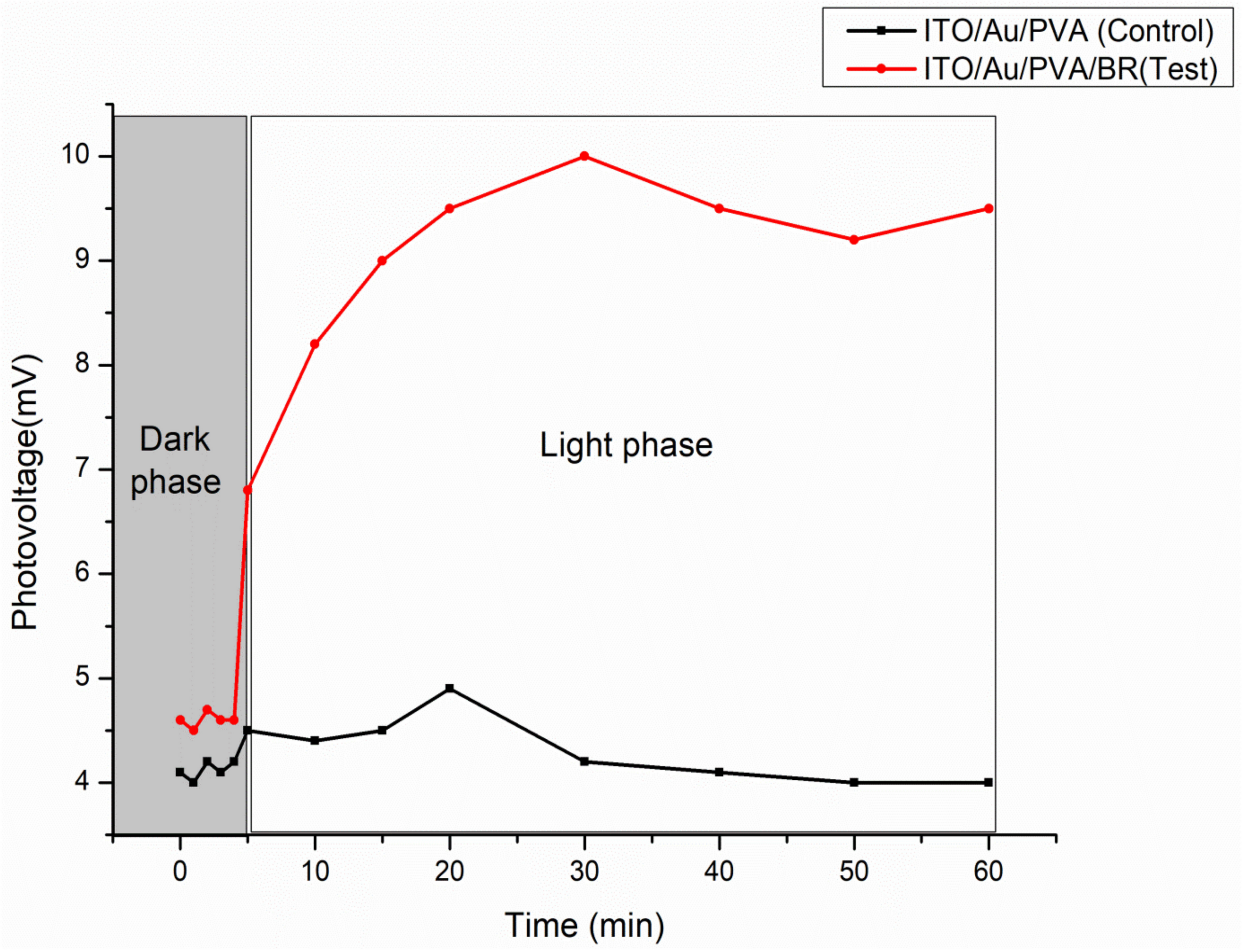
Photovoltage response of the bacteriorhodopsin based biosensor device under dark and light illumination conditions. Time dependent photovoltage measurement of the fabricated ITO/Au/PVA/BR device (test) compared with the ITO/Au/PVA assembly without protein (control).

The photoresponsiveness and proton transfer efficiency of purified *Halostagnicola larsenii* were analyzed using a fabricated ITO/Au/PVA/BR biosensor assembly. The schematic representation of fabricated biosensor assembly was shown in Figure 12. As shown in Figure 13, the device performance was monitored under altering dark and light phases to characterize its optoelectronic properties. During the initial dark phase (0–5 min), the device exhibited stable baseline negative photovoltage of approximately 4.0–4.5 mV. The observed voltage represents the intrinsic electrical response of the sandwich structured assembly in the absence of illumination. This negative photocurrent attributed to the intrinsic capacitive response of the sandwich-structured assembly in the dark or by small background electrochemical fluctuations. Upon light illumination (1,500 lux; 5–60 min), a rapid increase in photo-voltage was observed in the BR coated device compared to the control assembly lacking BR. The photovoltage increased sharply right after light exposure, reached at maximum of 10 mV. This demonstrates the efficient photo-activation of BR molecules that are immobilized within the PVA matrix. The observed light-induced electrical response confirms the functional activity of BR as a light-driven proton pump. Photo-excitation of BR facilitates efficient proton separation across the bio-interface, translating biological proton motive force into a detectable and stable electrical output.

## Discussion

### Isolation of haloarchaea and quantification of bacteriorhodopsin

Microbial communities thriving under extreme conditions possess unique metabolic potential that can be bio-prospected for industrial uses. In this study, Haloarchaeal strains were isolated from the salt pans of Tuticorin, identified, and screened for BR production. A total 10 haloarchaeal strains were isolated, of which eight strains showed pigmentation. Pigmentation in haloarchaeal strains typically indicates the presence of C50 carotenoids, popularly known as bacterioruberins, which impart characteristic pink-red hues to the colonies (Grant, 2001). Consistent with this, the pigmented isolates obtained in this study showed a similar pink to reddish coloration. Majority of the isolates obtained in this study (*n* = 7) were Gram negative. This observation aligns with existing reports that the majority of haloarchaea are Gram-negative, although certain coccoid species such as *Halostagnicola*, *Natronococcus*, and *Halococcus* can exhibit Gram variability within the order Halobacteriales (Oren et al., 1997). Morphological examination indicated that the isolates displayed pleomorphism, a common feature among haloarchaea. In liquid culture, these organisms exhibit motile rod and pleomorphic forms; while on solid media, they generally appear as non-motile cocci (Oren et al., 1997).

The isolate *Halostagnicola larsenii* (TP6) produced maximum concentration of BR (360.65 ± 0.9 mg/L) within their cells under elevated salinity (3.4 M) and low oxygen levels reflects adaptive metabolic regulation in haloarchaea. Elevated salinity levels promote the development of purple membrane structures, which are abundant in BR, thus bolstering membrane stability and ionic balance. Under oxygen scarce conditions, haloarchaea transition from aerobic respiration to light-dependent energy generation, utilizing BR as a proton pump, to create motive force and produce ATP, independently of oxygen respiration (Thombre et al., 2016). Thus the combined impacts of hypersalinity and limited oxygen availability increase BR production as an environmental adaptation strategy, explaining the higher intracellular BR accumulation observed in TP6. The concentration of BR produced by *Halostagnicola larsenii* notably surpasses the previously reported maximum of 250 mg/L for cultivable *Halostagnicola larsenii* by Kanekar et al. (2015), highlighting the significance of the wild strains isolated from unique hypersaline environments. Such native isolates possess a naturally enhanced BR production rate compared to previously characterized laboratory strains, underscoring the ecological and biotechnological value of untapped environmental microbiota. Such high production underscores its promise for scalable production and potential industrial applications, reducing the dependence on complex downstream processing. The high BR yield and phenotypic characteristics of the *Halostagnicola larsenii* (TP6) isolated in this study are comparable to those of other haloarchaea, identified from a saltwater lake in Inner Mongolia, China which are obligately halophilic, producing reddish-pink colonies (Castillo et al., 2006). This finding also aligns with earlier studies highlighting the potential of halophilic archaea as promising candidates for membrane-bound photoreceptive protein production (Antunes et al., 2016), given their enhanced BR productivity. Further characterization and purification of BR from *Halostagnicola larsenii* were subsequently carried out to assess its structural and functional attributes.

### Molecular identification of isolated haloarchaea

The present study reports the occurrence of Haloarchaeal strains from the Indian saltpans with higher salinity levels (20–25% w/v) compared to the hypersaline lakes like Lake Xilinhot in Inner Mongolia, China (Castillo et al., 2006). Molecular identification of the haloarcheal isolates based on 16 rRNA sequencing revealed a considerable taxonomic diversity within the Tuticorin salt pan, encompassing three genera, *Halococcaceae*, *Halobacteriaceae*, and *Halostagnicola*, reflecting a wide phylogenetic diversity of BR producing haloarchaea. This observation is consistent with previous studies reporting saltpans as reservoirs of diverse halophilic archaea adapted to extreme salinity (Oren et al., 2005; Ventosa et al., 2012). The present study represents the first investigation exploring BR production and pigment characterization in a native *Halostagnicola larsenii* strain from a hypersaline saltpan habitat. Isolates exhibiting ≥ 98% 16S rRNA similarity may display distinct phenotypic and functional traits; the high BR yield observed in *Halostagnicola larsenii* likely reflects adaptions to natural environmental conditions (Neagu and Stancu, 2025). The identification of TP2 as an uncultured halophilic archaeon emphasizes the unexplored microbial diversity in saltpan and their potential as sources of novel extremophiles with biotechnological properties.

### Optimization of growth and Bacteriorhodopsin production conditions

The optimization study suggested that supplementation of exogenous carbon sources negatively impacted BR production in *Halostagnicola larsenii* compared to the control minimal saline medium (360.65 ± 0.9 mg/mL). it indicates that BR biosynthesis is not dependent on exogenous carbon availability. Eventhough starch at 5% concentration yield the highest Br production among all carbon sources tested (Approx 130 mg/L), this remained substantially elevated than native conditions. It suggests that complex carbon sources are only partially tolerated. The negative impact of 1% sucrose on BR production and growth observed here parallels earlier studies that emphasize the importance of carbon source concentration in modulating metabolite synthesis (DasSarma and DasSarma, 2015). Carbon sources indirectly influence haloarchaeal growth; nevertheless, light- dependent energy metabolism under nutrient limiting conditions;rather than carbon availability, is the primary regulator of BR synthesis (Janos, 2004).

Furthermore, high salinity concentration in cultivation medium inherently limits microbial contamination thus lowering sterilization and operational expenses and improving economic feasibility. In support of the present findings, the type strain of *Halostagnicola larsenii* XH 48^T^, which shares phenotypic and physiological characteristics with our isolate TP6, has been reported to utilize a wide range of carbon sources for growth (Castillo et al., 2006), yet our findings demonstrate that such utilization does not translate to enhanced BR biosynthesis in TP6 isolate.

The nitrogen source, KNO_3_ had no significant effect on growth and BR yield of *Halostagnicola larsenii* which can be attributed to the presence of yeast extract in the minimal saline medium, which supplies amino acids, peptides, and growth factors that can be readily utilized. Under such nutrient–rich conditions, nitrogen is unlikely to be limiting, and haloarchaea preferentially utilize organic nitrogen while repressing energy-intensive nitrate assimilation pathways. These results are consistent with the findings of Khanafari et al. (2010), who demonstrated that organic nitrogen sources such as yeast extract and peptone are superior to inorganic forms for supporting both biomass growth and metabolite synthesis in halophilic archaea. Combining multiple organic nitrogen sources can further enhance biomass and BR yield compared to using a single nitrogen source (Ghasemi et al., 2008). The negative impact of nitrogen supplementation on growth may be attributed to osmotic stress induced by nitrogen compounds in an already salinity rich medium, interfering the ionic balance important for halophilic archaeal metabolism (Oren, 2013). This indicates that nutrient composition plays a decisive role in regulating BR biosynthesis, suggesting that BR synthesis in *Halostagnicola larsenii* TP6 functioning as a stress adaptive phototrophic mechanism triggered under nutrient limiting conditions (Fendrihan et al., 2006) . Collectively, these findings reveal that nutrient composition plays a conclusive part in regulation BR biosynthesis, and maximum BR yield in *Halostagnicola larsenii* achieved by the use of minimal saline medium and optimized culture conditions.

### Yield analysis and purification efficiency of bacteriorhodopsin

In this study, bead mix (BM) method coupled with a modified lysis buffer yield maximum BR from *Halostagnicola larsenii* TP6 and improved BR recovery, compared to the conventional freeze-thaw (FT) technique. The cell lysis buffer disrupts the cells releasing the integral membrane proteins into the cell lysate solution. The combined mechanical shear and collision forces generated during bead agitation resulted in effective cell disruption and enhanced membrane protein release, in line with previous findings by Oesterhelt and Stoeckenius, (1971). Evidently, the high-speed centrifugation during the BM preparation effectively removed contaminants from the cell lysate, improving BR purity (Hsu et al., 2013). Traditionally, BR can be pelleted from cell lysates of *Halobacterium salinarum* by high-speed centrifugation, effectively separating the membrane fraction from nucleic acids, proteins, and soluble contaminants. However, its limitations include a lengthy processing time, high equipment cost, and reduced yield (∼70%) due to the inherent difficulty in harvesting the delicate purple band formed during centrifugation (Yatsunami et al., 2000). In contrast, the bead mix method combined with an aqueous two-phase purification system (ATPS) followed in this study enabled BR extraction and purification within 3 h, offering a rapid, scalable, and efficient alternative. We also acknowledge that the recovery of maximum BR yield with improved purity resulted from the incorporation of CHAPS detergent, which ensured the effective membrane disruption. This substantial retention of BR content after purification suggests that the BM method is well-suited for downstream processing and scale-up for industrial applications. Furthermore, adoption of an aqueous two-phase purification method after BM extraction proved advantageous for rapid purple membrane isolation, bypassing the time, cost, and recovery limitations associated with sucrose gradient ultracentrifugation (SGU). The observed band at 26 kDa was characteristics of BR, similar to BR at previous reports (Shiu et al., 2014)

This synergistic effect of selecting a high-yield strain *Halostagnicola larsenii* and employing a high recovery BM based purification method significantly improves the overall productivity. The bead mix approach followed by aqueous two-phase purification yielded a moderate BR concentration with good total yield, offering high lysis efficiency and functional BR retention. These findings indicate that although initial purity was lower in the crude extracts, the BM method still resulted in a higher post-purification BR yield, supporting its suitability for efficient recovery and downstream processing. Compared to other extraction methods, the BM-based approach provides a considerably efficient recovery, which could be advantageous for applications requiring high-yield BR production. Although aqueous three-phase and microfluidic systems demonstrated higher recovery or rapid separation, their limitations- including lack of functional validation, structural instability risks, and poor scalability- limit their broader applicability (Table 4).

**TABLE 4:** Comparison of various bacteriorhodopsin (BR) extraction and purification methods based on yield, purity, extraction efficiency, host strain and limitation of both conventional and advanced techniques.

Extraction method	BR content (mg/L)	Efficiency	Strain	Limitation	References
Bead mixing followed by Aqueous two-phase system purification	48.4	High lysis efficiency: retains functional BR : less time: scalability	*Halostagnicola larsenii* TP6	Limited validation	Current study
Aqueous two-phase system preparation	38.6	fast and facile purification; scalable	*Halobacterium salinarum*	Limited validation; Emulsion Risk	(Shiu et al., 2013)
Aqueous-three-phase system	55.9	High recovery (∼90%) and reasonably good purity.	*Halobacterium salinarum*	Risks altering BR’s functional or structural properties	(Shiu et al., 2014)
ATPS and Microfluidic separation system	50	Very rapid, low sample consumption	*Halobacterium salinarium* R1 (ATCC 29341)	Lengthy process: Low scalability: Device complexity	(Huh et al., 2010)
Sucrose density gradient ultracen trifugation (SGU)	37.6	BR with a higher purity	*Halobacterium halobium NRL*	Lengthy process.	(Oesterhelt and Stoeckenius, 1974)
BR was extracted suing Triton X-100 detergent	0.372	Higher stability	*Halobacterium holobium* JW-3	Lengthy process; low scalability;	(Reyenolds and Stoeckenius, 1977)

### Characterization of bacteriorhodopsin

The presence of absorbance peak at 550 nm attributed to the presence of BR in the purified sample according to previous reports (Alsafadi et al., 2018). The UV-Vis absorbance profile and TLC analysis collectively validated the successful isolation of native BR from *Halostagnicola larsenii*. The UV-Vis absorption spectra of purified BR samples obtained from different extraction processes. The consistency between the native and standard BR in both absorbance characteristics and chromatographic behavior underscores the structural similarity of the isolated protein to commercially available BR (Fanaei and Emtiazi, 2014). The minor variation observed between the Rf values can be attributed to slight differences in sample concentration, purity levels, or micro environmental factors during chromatographic migration, which are commonly acceptable.

Raman spectroscopic analysis of the *Halostagnicola larsenii* isolate exhibited a prominent Raman shift at 1530 cm^−1^ corresponding to the V1 (C = C) stretching band, a major characteristic feature of the BR chromophore structure. This distinctive peak strongly indicates the presence and structural integrity of BR in the sample, confirming its purity. The result aligns closely with previously established data for BR (Alsafadi et al., 2018; Figure 10). Consistent with earlier reports, the current study thus confirm that *Halostagnicola larsenii* one of the potent producers of BR, and it’s validated through resonance Raman spectroscopy. The correlation between the effective conjugated chain length and the Raman shift (cm^–1^) of the V1 band has been shown in earlier research (Smith, 2010). This association enables the identification of double bonds in polyconjugated main chains of carotenoids, such as lycopene, β-carotene, dodecapreno-β-carotene, and retinal. Thus, a key marker band for elucidating the structure of bacterioruberin is the V1 (C=C) stretching mode. It also provides non-destructive, label-free molecular insights into protein and functional groups confirmation, making it a valuable tool for assessing structural integrity and purity of BR.

BR is a seven-transmembrane, hydrophobic protein that is linked to the retina via a Schiff base bond with a lysine residue. It is especially prone to conformational changes and fragmentation during chromatographic analysis under denaturing conditions because of its membrane-associated nature and covalent retinal attachment (Yuan et al., 2018; Oesterhelt and Stoeckenius, 1974). In the protein structure, amino acids like tyrosine and tryptophan are especially targeted by 280 nm detection in reverse- phase HPLC. It is unclear that the retina can be directly detected at this wavelength unless it is covalently attached or connected with the protein matrix, as its maximal absorbance found between 360 and 380 nm (Zhang et al., 2011). Therefore, the minor peaks in the chromatogram might represent various BR conformational states during the denaturation and separation process. In reverse-phase HPLC investigations of various membrane proteins, where partial unfolding or proteolytic cleavage produces different elution profiles, similar multi-peak patterns have been documented (Hampp, 2000). According to earlier research using reverse-phase HPLC for the characterisation of microbial rhodopsins, the noticeable peak at 2.91 min verified the successful enrichment of BR (Ram-Mohan et al., 2016). Further confirmation through multi-wavelength detection or mass spectrometry would be required to conclusively identify the molecular nature of these peaks. The combined spectroscopic, chromatographic, and Raman analyses collectively confirmed successful purification of functionally intact BR.

### Assessment of bacteriorhodopsin for photoelectrochemical sensing

Light induced proton transport across the purple membrane of BR causes charge separation and the subsequent production of photocurrent (Cao et al., 2015). In the present study, purified BR obtained from *Halostagnicola larsenii* was integrated into an ITO/Au/PVA biohybrid assembly to evaluate its photoelectrochemical behavior. The fabricated device demonstrated stable photovoltage generation following light illumination, whereas minimal response was measured during the dark phase. Control measurements using ITO/Au/PVA without BR confirmed that the enhanced photovoltage generated primarily from BR photo-activation rather than electrode artifacts or capacitive background effects. The steady and reliable photovoltage plateau seen under continuous illumination shows the effective stabilization of the PVA matrix with minimal protein hydration and structural integrity (Patil et al., 2012).

The photoelectrochemical measurements reported in this study were performed using the PVA immobilized BR layer. Polyvinyl alcohol (PVA) film was employed as a supportive, hydrophilic matrix for BR immobilization. The incorporation of a PVA membrane aimed to enhance the durability, hydration retention, and mechanical stability of the BR layer, thereby maintaining stable photocurrent generation. Compared with any existing protein based biosensors that suffer from rapid dehydration and loss of activity, the PVA matrix enhances operational stability and enables repeated use under ambient conditions. This stabilization strategy represents a practical advantage over conventional bioelectronics devices where protein denaturation limits long term performance (Kanekar et al., 2021).

Compared with conventional enzyme based biosensors, extremophilic BR based biosensor systems offer several intrinsic advantages, such as photostability, reversible photoactivity, low energy consumption, and resistance to harsh environmental conditions (Mostafa and Elfiki, 2024). Current biosensor markets are largely dominated by enzymatic sensing platforms, which are widely used in medical diagnostics, environmental monitoring, and food sector. These enzyme based sensing platforms, require cofactors or chemical substrates and have limited operational stability (Herrera-Domínguez et al., 2023; Yildiz and Koç, 2023). BR based biosensor operate through light driven proton pumping activity, enabling rapid signal generation with minimal external energy input and reduced operational cost compared to conventional biosensing systems (Bondar and Smith, 2025). These characteristics have stimulated interest in the application of BR in light driven biochips and bio-optoelectronic sensors that are commonly used in the food industries and other biotechnological applications (Wu et al., 2012). Recent studies demonstrated the incorporation of BR into bio-electronic transistors and photonic sensing platforms, highlighting its potential positioning within sustainable biosensing, bio-electronic, and renewable bioenergy assisted sensing markets (Huang et al., 2025; Li et al., 2024).

In the present study, the prototype demonstrated dependable photo-stability and photo-response of BR isolated from *Halostagnicola larsenii*, underscoring its potential for application in diagnostic fields. BR served as the photoactive transducer enabling light driven signal amplification upon pathogen binding for medical diagnostic applications. The high physicochemical stability and light driven photo activity of BR make it suitable transducer for label free and reusable biosensing platforms. Functional validation through system level integration and long term performance testing is essential before advancing toward practical deployment. BR derived from haloarchaeal extremophiles is gaining attention for emerging applications in bio-optoelectronics, optical data storage, artificial vision system, and light driven biosensors (Wu et al., 2025). Therefore, the identification of high yield BR producing *Halostagnicola larsenii* provides a valuable resource for future biotechnological and sustainable bioelectronics applications.

## Conclusion

This study represents the first systematic and comprehensive report of BR producing haloarchaea from the largest salt pan in Tuticorin, Tamil Nadu. Among the strains isolated, *Halostagnicola larsenii* demonstrated the highest BR production (360.65 ± 0.9 mg/L) during pigment screening. To our best knowledge, this represents the first report of a BR producing *Halostagnicola larsenii* strain isolated from an Indian salt pan ecosystem. Notably, the BR productivity of *Halostagnicola larsenii* was comparatively higher than the yields typically reported for haloarchaeal strains isolated from other natural habitats. Supplementation of carbon and nitrogen sources negatively impacted both growth and BR production in *Halostagnicola larsenii*, confirming that native minimal saline requirements with optimized culture conditions are optimal for maximum BR biosynthesis The intracellular BR was extracted from haloarchaea by a cost effective, easy, efficient, and environmentally friendly bead mix method, yielding maximum protein (72.7%) compared to the conventional extraction methods. These findings underscore the dual importance of strain selection and purification strategies in maximizing BR yield. BR characterization using Raman spectroscopy, and SDS-PAGE, confirmed that the BR produced by *Halostagnicola larsenii* has features similar to previously reported BR producing haloarchaeal strains. The chromatographic profile observed in this investigation highlights the relevance of RP-HPLC in determining the molecular integrity and purity of hydrophobic membrane proteins. Additionally, the observed photocurrent activity of isolated BR indicates its potential use in the development of biosensor for industrial applications. Overall, this work highlights the ecological and biotechnological significance of salt pans as a natural microbiological reservoir for industrially important compounds like BR. Continued research and development in this area promise to unlock further potential in various fields and foster sustainable technological innovations.

## Data Availability

The datasets presented in this study can be found in online repositories. The names of the repository/repositories and accession number(s) can be found in the article/Supplementary material.
