# Supplementary material for: Extremophilic bacteriorhodopsin from hypersaline salt pan: characterization and photoelectrochemical assessment for potential biosensor applications
Source: Front Microbiol. 2026 Jun 4;17:1805566. doi: 10.3389/fmicb.2026.1805566 (PMC13275480; doi:10.3389/fmicb.2026.1805566)
Supplement: Supplementary file 1 [file Supplementary_file_1.docx]

**Supplementary material**

1. **Methodology section**
   1. **Composition of minimal saline medium(per liter)** : Casamino acid – 0.075 g, Yeast extract – 1 g, Na_3_C_6_H_5_0_7_ – 3 g, KCl – 2 g, MgSO_4_ – 20 g, FeSO_4_ – 0.05 g, MnSO_4_ – 0.2 g, 5% NaCl- 250 g). The pH and salinity of the medium was adjusted to 7.2 and 3.42 M NaCl (20% NaCl (w/v) respectively.pH 7.2
   2. **Methodology for quantification of bacteriorhodopsin** : Two millilitre of the halo-bacterial culture was centrifuged at 12,000 rpm for 15 minutes at 4°C, suspended in 1ml of autoclaved deionised water provided with 30 µl of DNase (66 U/mL). From this, 900 µL of sample was analysed added to the separate tube followed by the addition of 50 µL of freshly prepared 4M NaOH and NH_4_OH in the dark condition. The absorbance of the samples was analysed immediately at 560 nm in a UV-Vis Spectrophotometer. To eliminate the retinal pigment present in the purple membrane, the samples were exposed to light (a process called bleaching) for 48 hours. After incubation under light (1500 lux) for 48 hours, the absorbance of the bleached samples was observed at 560 nm.
   3. **Instrument PCR cycle setup**: The initial denaturation at 95℃ for 5 minutes; followed by 35 cycles of denaturation at 94℃ for 30 sec, annealing at 58℃ for 45 seconds, extension at 72℃ for 45 seconds; and a final extension of 72 ℃ for 7 min and the mixture was held at 4℃.
   4. **The composition of minimal medium supplemented with carbon source (per liter):** Casamino acid – 0.075 g, Yeast extract – 1 g, Na_3_C_6_H_5_0_7_ – 3 g, KCl – 2 g, MgSO_4_ – 20 g, FeSO_4_ – 0.05 g, MnSO_4_ – 0.2 g, 5% NaCl- 250 g). The pH and salinity of the medium was adjusted to 7.2 and 3.42 M NaCl (20% NaCl (w/v) respectively. Glucose, Starch and Sucrose were supplemented separately at concentrations of 0.1 %( 0.05g), 1% (0.5g) and 5% (2.5g). pH 7.2
   5. **The composition of minimal medium supplemented with nitrogen source (per liter):** Casamino acid – 0.075 g, Yeast extract – 1 g, Na_3_C_6_H_5_0_7_ – 3 g, KCl – 2 g, MgSO_4_ – 20 g, FeSO_4_ – 0.05 g, MnSO_4_ – 0.2 g, 5% NaCl- 250 g). The pH and salinity of the medium was adjusted to 7.2 and 3.42 M NaCl (20% NaCl (w/v) respectively. KNO3 were supplemented separately at concentrations of 0.1 %( 0.05g), 1% (0.5g) and 5% (2.5g) pH 7.2

**Supplementary Figures**

**Figure 1.** Study area map**.**


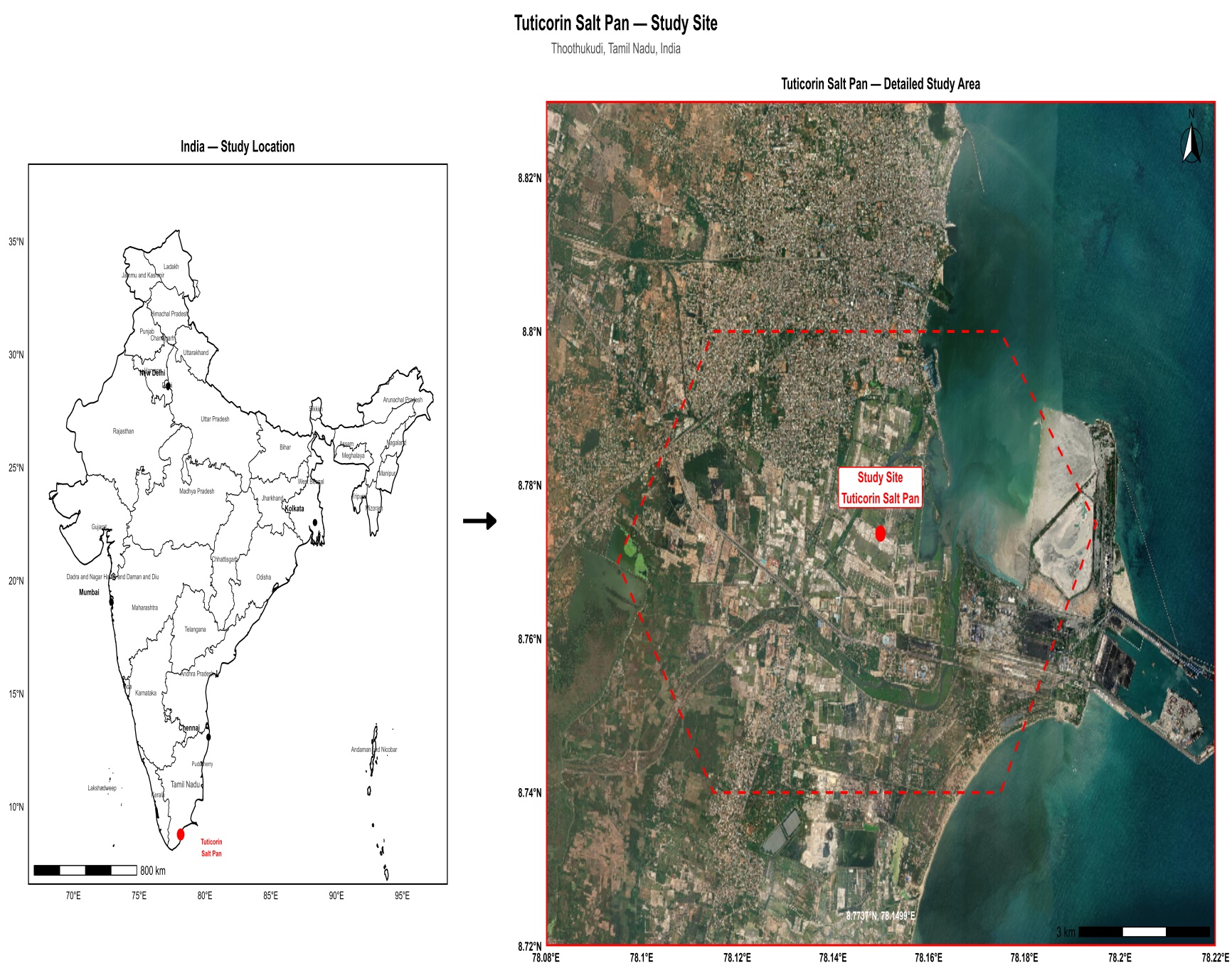


**Figure 2.** Halophilic archaeal isolates from Tuticorin saltpans, Tamil Nadu, India. (a) colony morphology of isolates displaying variable pigmentation ranging from light pink to orange-red on haloarchaeal minimal saline agar medium (20% (w/v) NaCl, pH 7.2) under 1500 lux light intensity. (b) slant cultures of isolates exhibiting characteristics carotenoid based pigmentation of BR producing haloarchaeal strains under controlled growth conditions.


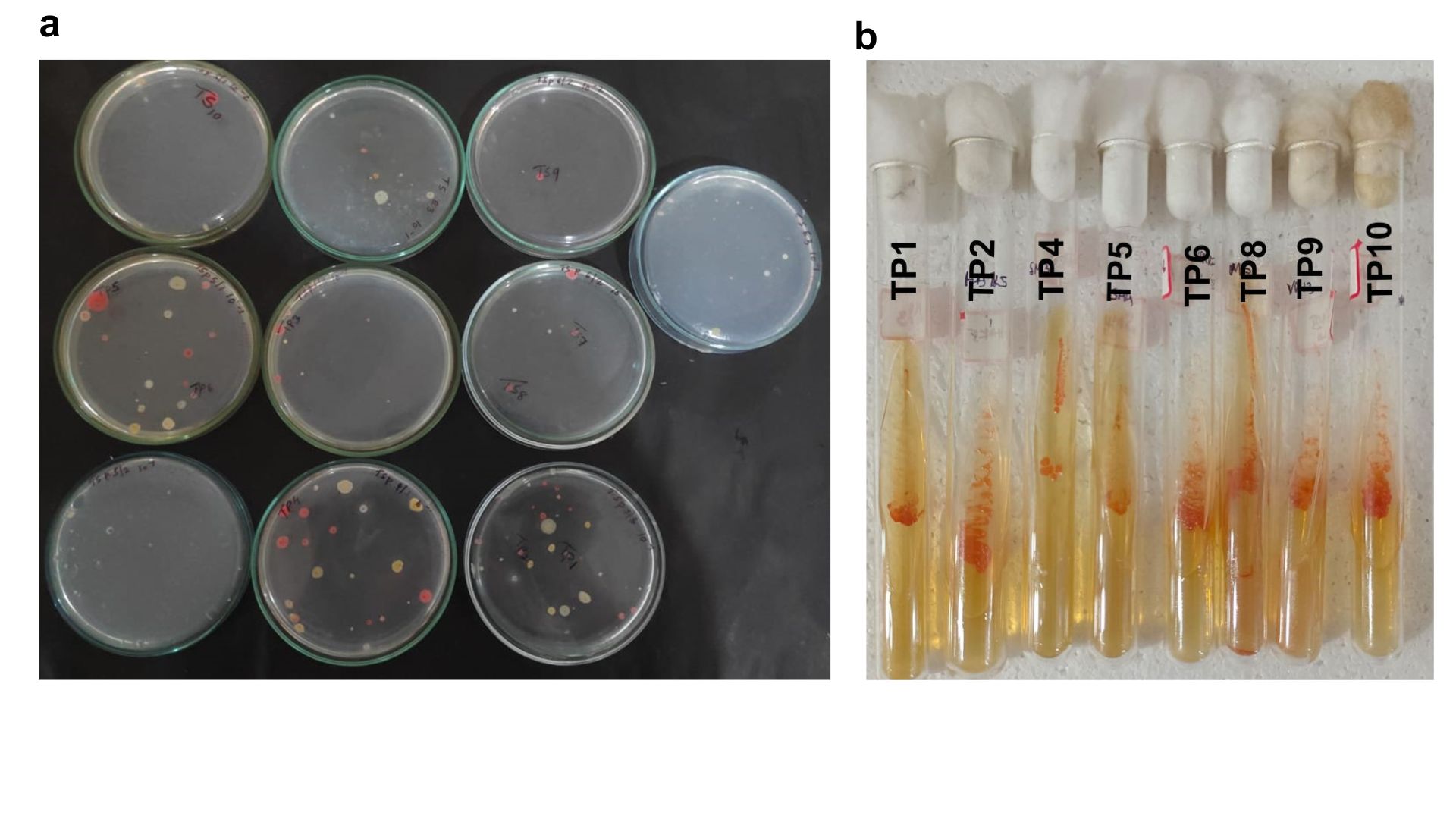


**Figure 3.** SDS-PAGE and UV-Vis analysis of BR extracted from TP6 strain.(a) SDS-PAGE profile of cell lysates obtained by two extraction methods: Lane 1- Freeze thaw method, Lane 2 - bead mix method and lane 3- Molecular marker and second gel picture showing the purified BR obtained by two extraction methods, with lines as described above.


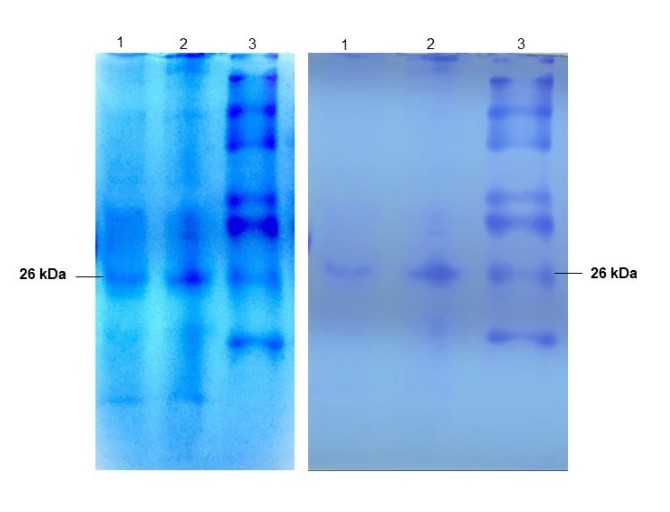


**Figure 4.** The TLC analysis of native BR from *Halostagnicola* sp. and commercial standard. (a) represent the native BR and (b) represent the standard.

**
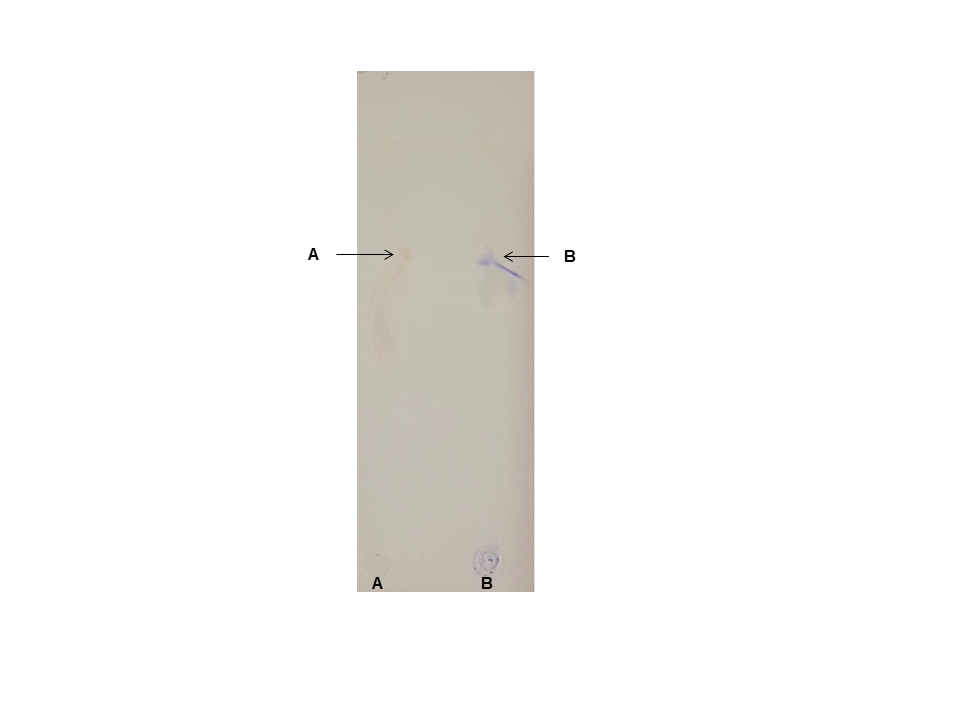
**

**Supplementary Tables**

**Table 1.** Summary of optimization results with the effect of carbon and nitrogen sources on the growth and BR production of TP6

| **Optimization Source** | **Concentration** | **Growth Response (10^8^ cells/mL)** | **BR Production (mg/L)** |
| --- | --- | --- | --- |
| **Control flask (without C or N sources)** | NA | 0.218 | 360.65 |
| **Carbon source** | | | |
| Glucose | 0.1% | 0.137 | 30.5 |
|  | 1% | 0.126 | 21.18 |
|  | 5% | 0.114 | 17.74 |
| Sucrose | 0.1% | 0.143 | 32.18 |
|  | 1% | 0.126 | 14.85 |
|  | 5% | 0.155 | 34 |
| Starch | 0.1% | 0.158 | 45.7 |
|  | 1% | 0.255 | 98.9 |
|  | 5% | 0.249 | 130.1 |
| **Nitrogen source** | | | |
| **KNO_3_** | 0.1% | 0.138 | 1.2 |
|  | 1% | 0.127 | 6.6 |
|  | 5% | 0.108 | 2.0 |

**Table 2:** Summary of the repeated measures of ANOVA analysis for differences in BR yield and bacterial abundance

| **Dependent variable** | **Source** | **Sum of Squares** | **Mean Square** | **F-value** | **p-value** | **Partial η**² |
| --- | --- | --- | --- | --- | --- | --- |
| Bacterial abundance | Carbon Source | 0.71 | 0.036 | 277.332 | <0.001 | 0.984 |
|  | Concentration | 0.003 | 0.001 | 10.094 | 0.005 | 0.692 |
|  | Carbon Source × Concentration | 0.019 | 0.005 | 37.243 | <0.001 | 0.943 |
|  | Nitrogen Source | 0.62 | 0.028 | 245.656 | <0.001 | 0.938 |
|  | Concentration | 0.002 | 0.004 | 12.167 | 0.005 | 0.735 |
|  | Nitrogen Source × Concentration | 0.013 | 0.004 | 36.478 | <0.001 | 0.925 |
| BR Yield | Carbon Source | 0.375 | 0.036 | 9.925 | 0.005 | 0.688 |
|  | Concentration | 0.087 | 0.043 | 2.3 | 0.156 | 0.338 |
|  | Carbon Source × Concentration | 0.160 | 0.040 | 2.112 | 0.162 | 0.484 |
|  | Nitrogen Source | 0.456 | 0.027 | 10.245 | 0.005 | 0.745 |
|  | Concentration | 0.002 | 0.005 | 3.723 | 0.187 | 0.423 |
|  | Nitrogen Source× Concentration | 0.016 | 0.046 | 35.673 | <0.001 | 0.936 |
